# Extracellular Vesicles in Osteogenesis: Comparative Analysis of Stem Cell Sources, Conditioning Strategies, and In Vitro Models Toward Advanced Bone Regeneration

**DOI:** 10.3390/cells15010027

**Published:** 2025-12-23

**Authors:** Luca Dalle Carbonare, Arianna Minoia, Michele Braggio, Francesca Cristiana Piritore, Anna Vareschi, Mattia Cominacini, Alberto Gandini, Franco Antoniazzi, Daping Cui, Maria Grazia Romanelli, Maria Teresa Valenti

**Affiliations:** 1 Department of Engineering for the Innovation Medicine, University of Verona, 37100 Verona, Italy; luca.dallecarbonare@univr.it (L.D.C.); arianna.minoia@univr.it (A.M.); anna.vareschi@univr.it (A.V.); mattia.cominacini@univr.it (M.C.); 2 Department of Neurosciences, Biomedicine and Movement Sciences, University of Verona, 37100 Verona, Italy; michele.braggio@univr.it (M.B.); francescacristiana.piritore@univr.it (F.C.P.); mariagrazia.romanelli@univr.it (M.G.R.); 3 Department of Surgery, Dentistry, Paediatrics and Gynaecology, University of Verona, 37134 Verona, Italy; alberto.gandini@univr.it (A.G.); franco.antoniazzi@univr.it (F.A.); 4 Department of Orthopedics, Shenzhen Bao’an District Central Hospital, Shenzhen 518100, China; dapingcui@gmail.com

**Keywords:** extracellular vesicles, bone, mesenchymal stem cells, tissue regeneration

## Abstract

Extracellular vesicles (EVs) derived from stem cells have emerged as promising mediators of osteogenesis, suggesting cell-free alternatives for bone tissue engineering and regenerative medicine. This review provides a comprehensive analysis of the main stem cell sources used for EV production, including bone marrow mesenchymal stem cells (BM-MSCs), adipose-derived stem cells (ADSCs), umbilical cord MSCs (UC-MSCs), induced pluripotent stem cells (iPSCs), and alternative stromal populations. Particular attention is given to the ways in which different conditioning and differentiation strategies, such as osteogenic induction, hypoxia, and mechanical stimulation, modulate EV cargo composition and enhance their therapeutic potential. We further discuss the in vitro models employed to evaluate EV-mediated bone regeneration, ranging from 2D cultures to complex 3D spheroids, scaffold-based systems, and bone organoids. Overall, this review emphasizes the current challenges related to standardization, scalable production, and clinical translation. It also outlines future directions, including bioengineering approaches, advanced preclinical models, and the integration of multi-omics approaches and artificial intelligence to optimize EV-based therapies. By integrating current knowledge, this work aims to guide researchers toward more consistent and physiologically relevant strategies to harness EVs for effective bone regeneration. Finally, this work uniquely integrates a comparative analysis of EVs from multiple stem cell sources with engineering strategies and emerging clinical perspectives, thereby providing an updated and translational framework for their application in bone regeneration.

## 1. Introduction

Bone tissue regeneration and homeostasis rely on a complex interplay of cellular signals that coordinate osteogenesis, the process by which new bone is formed. Among the key regulators of this process are mesenchymal stem cells (MSCs) and related progenitor cells, which contribute to bone repair not only through direct differentiation but also via the secretion of extracellular vesicles (EVs) [1].

It is well known that EVs are membrane-bound nanoparticles that carry a bioactive cargo including RNAs, proteins, and lipids capable of modulating the functions of recipient cells [2,3]. The MISEV2018 and updated MISEV2023 guidelines, issued by the International Society for Extracellular Vesicles (ISEV), provide a standardized framework for working with extracellular vesicles [4]. Accordingly, EVs comprise heterogeneous subpopulations that differ in terms of biogenesis, size, molecular cargo, and functional relevance [4,5]. Exosomes (30–150 nm) originate from the endosomal pathway through inward budding of multivesicular bodies (MVBs) and are released upon fusion with the plasma membrane. Cargo loading involves Endosomal Sorting Complex Required for Transport (ESCRT)-dependent or ESCRT-independent mechanisms, contributing to their specialized roles in cell–cell communication and therapeutic delivery [6].

Microvesicles (100–1000 nm) form by outward budding of the plasma membrane and carry cytosolic and membrane-derived cargo that reflects rapid cellular responses and local microenvironmental cues [4,7].

Apoptotic bodies (500–2000 nm) arise during programmed cell death and contain fragmented DNA, organelles, and cytosolic components; although once considered cellular debris, they are now recognized as modulators of immune and regenerative processes [4,8]. The category of small EVs (sEVs) encompasses vesicles < 200 nm independent of their biogenesis pathway, following the recommendations of the MISEV2018 guidelines [4]. Beyond nomenclature, the guidelines outline minimal requirements for EV isolation, recommending established approaches such as differential ultracentrifugation, density-gradient ultracentrifugation, size-exclusion chromatography, ultrafiltration, and polymer-based precipitation. They highlight that combining complementary techniques—such as ultracentrifugation with size-exclusion chromatography—can improve vesicle purity and reduce the co-isolation of protein aggregates, lipoproteins, or cell debris. Regarding EV characterization, the guidelines emphasize the need to verify vesicle identity through the detection of canonical EV-associated markers (e.g., tetraspanins such as CD9, CD63, CD81; ESCRT-associated proteins such as TSG101 and Alix) and the demonstration of the absence of non-EV contaminants (e.g., GM130 for Golgi, calnexin for ER, or ApoA1 for lipoproteins). Characterization should also include the assessment of particle size and concentration using complementary methods such as nanoparticle tracking analysis, tunable resistive pulse sensing, electron microscopy, or atomic force microscopy. Furthermore, the guidelines stress the transparent reporting of all experimental parameters, including detailed descriptions of isolation protocols (centrifugation speed, time, rotor type; chromatography column specifications), vesicle quantification methods (protein content, particle number, lipid quantification), and functional assays used to evaluate the biological activity. Clear documentation of controls, dose metrics, and normalization strategies (e.g., particle number, protein mass, or cell equivalents) is essential to ensure reproducibility and comparability across studies.

Despite these distinctions, the terminology used across studies is often inconsistent, and many experimental systems do not allow clear separation of EV subtypes. Therefore, in this review, we use the general term “EVs” when discussing findings, unless the original studies explicitly discriminate between specific vesicle types, directing the reader to those primary sources for further mechanistic clarification. In recent years, EVs have emerged as critical mediators of intercellular communication in bone biology, with several studies demonstrating their ability to modulate osteogenic differentiation, promote matrix mineralization, and regulate inflammation within the bone microenvironment [9]. Importantly, the osteoinductive potential of EVs appears to be closely dependent on their cellular origin, as well as on the physiological or pre-differentiation state of the parent cells. For this reason, comparative studies analyzing EVs derived from various stem cell sources (e.g., bone marrow mesenchymal stem cells (BM-MSCs), adipose-derived stem cells (ADSCs) and induced pluripotent stem cells (iPSCs)) have gained increasing attention in the context of bone regeneration.

Moreover, the differentiation strategies adopted to induce osteogenesis in donor cells ranging from chemical induction to mechanical stimulation or hypoxia have been shown to influence the EV cargo composition and, consequently, their biological activity [10]. This opens up useful possibilities for engineering EVs with enhanced osteogenic potential through preconditioning or tailored culture systems.

However, to better assess the function of EVs in promoting osteogenesis, novel in vitro models are also being explored. Three-dimensional systems and, in particular, bone organoids represent a promising tool for studying complex multicellular interactions and spatially organized tissue development [11]. These models provide a more physiologically relevant environment compared to traditional 2D cultures and may serve as platforms for testing EV-mediated effects on bone formation and remodeling.

**Figure 1:**
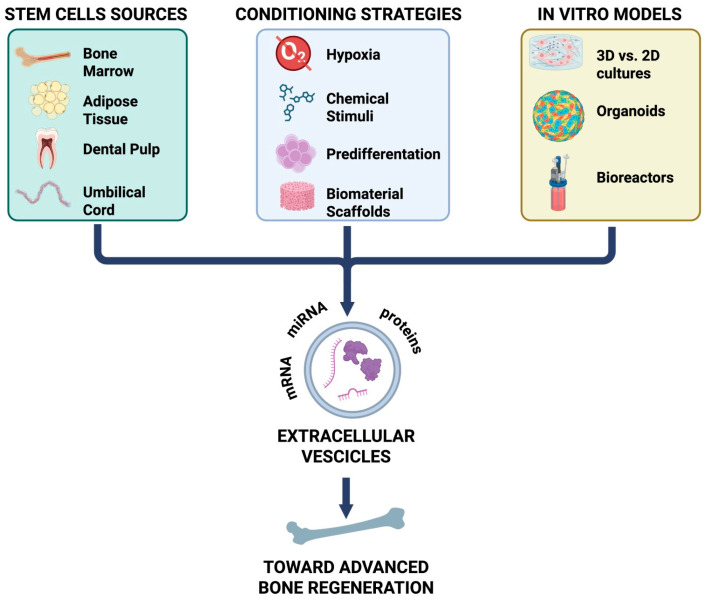
Mesenchymal stem cells (MSCs) derived from various tissue sources (bone marrow, adipose tissue, dental pulp, and umbilical cord) release EVs, whose content and function are shaped by different conditioning strategies (hypoxia, chemical stimuli, pre-differentiation, and biomaterial scaffolds). In vitro models, including 2D versus 3D cultures, organoids, and bioreactors, provide platforms to study these effects. Collectively, EVs enriched in proteins, miRNA, and mRNA contribute to advanced bone regeneration. mRNA: messenger ribonucleic acid; miRNA: micro-RNA.

In this review, we aim to provide a comprehensive overview of the current knowledge on EVs in osteogenesis, with a focus on the comparing EVs derived from different stem cell sources, evaluating the impact of various differentiation strategies on EV bioactivity and exploring the potential of bone organoids as next-generation platforms to study EV function in a controlled and biomimetic context (Figure 1).

Finally, in this review, we extend beyond previous overviews by (1) providing a comparative analysis of EVs derived from multiple stem cell sources and outlining their relative advantages and limitations for bone repair; (2) integrating emerging conditioning and engineering approaches with both in vitro and in vivo evidence; and (3) dedicating a focused discussion on current and potential clinical applications.

## 2. Osteogenesis

**Figure 2:**
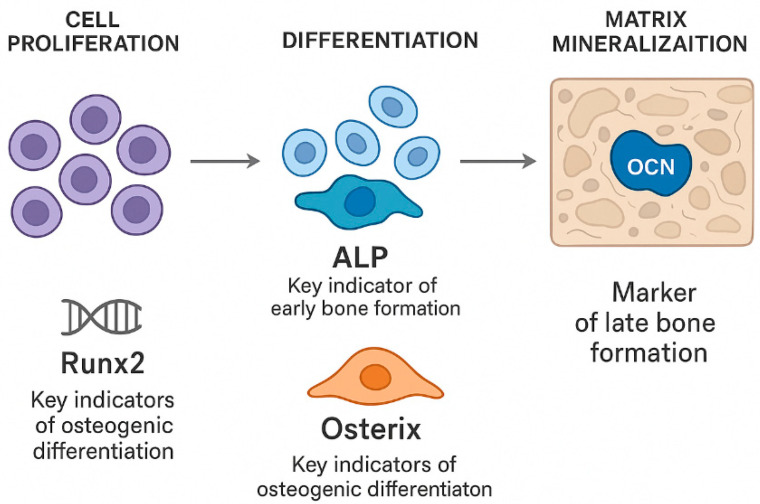
The process of bone formation consists of three closely interconnected stages: cellular proliferation, differentiation, and matrix mineralization. Osteoblasts, primarily derived from BM-SCs, play a central role throughout all phases of new bone formation. The transcription factors RUNX2 and OSX are essential for regulating osteoblastic differentiation: increased expression of these transcription factors promotes the maturation of osteogenic cells, whereas their absence impairs osteoblast development.

It is well known that bone formation progresses through three interconnected stages: cell proliferation, differentiation, and matrix mineralization (Figure 2) [12]. Osteoblasts, which mainly arise from BM-MSCs with strong osteogenic potential, are central to this process [13]. A key indicator of early bone formation is the bone-specific enzyme alkaline phosphatase (ALP), whose expression closely reflects the level of osteoblast differentiation. As the ALP activity increases, pre-osteoblasts more effectively mature into fully developed osteoblasts, highlighting the enzyme’s role in regulating the transition from immature to mature bone-forming cells [14]. Moreover, osteocalcin (OCN) is a bone-specific protein primarily produced and secreted by osteoblasts, with the majority being incorporated into the bone matrix, making it a well-recognized marker of the late stage of bone formation [14]. Furthermore, both runt-related transcription factor 2 (RUNX2) and Osterix (OSX) serve as key indicators of osteogenic differentiation [15]. Increased expression of OSX facilitates the progression of osteogenic differentiation, whereas its absence leads to a deficiency of osteoblasts, thus underscoring its essential role in bone development [15].

## 3. Stem Cell-Derived EVs in Osteogenesis

This section presents a comparative overview of the most commonly used stem cell sources for generating EVs with osteogenic activity. We explore the unique properties of EVs derived from BM-MSCs, ADSCs, umbilical cord MSCs (UC-MSCs), iPSCs, and alternative stromal populations, providing a synthesis of the current evidence and highlighting key differences in their regenerative efficacy.

**Figure 3:**
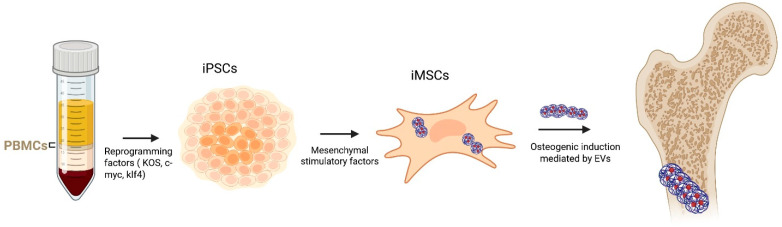
PBMCs are reprogrammed using pluripotency factors (KOS, c-Myc, and Klf4), which are then differentiated into iMSCs under mesenchymal stimulatory conditions. By inducing osteogenesis, the EVs derived from iMSC promote osteoblast formation. Arrows indicate the sequential flow of the reprogramming, differentiation, and osteogenic processes.

Recent studies have shown that EVs derived from different stem cell sources, such as BM-MSCs, ADSCs, UC-MSCs, and dental or synovial stem cells, exhibit heterogeneous molecular profiles and distinct osteogenic capacities [16,17,18]. Several studies have demonstrated that BMSC-EVs play a crucial role in bone formation by transporting specific miRNAs, making them a promising therapeutic strategy for bone metabolic disorders [19]. Compared to MSCs obtained from bone marrow or other tissues, ADSCs offer several notable benefits. In fact, these cells are easier to obtain and to collect; they are more plentiful, and their isolation involves reduced donor-site injury [20]. In addition, UC-MSCs-EVs are particularly attractive for therapeutic applications because they can be obtained non-invasively from discarded clinical specimens, exhibit rapid proliferation, display low immunogenicity, and avoid ethical concerns [21]. Moreover, through the use of gene reprogramming technology, adult cells can be reprogrammed into iPSCs, a unique cell population that can undergo dedifferentiation, in order to differentiate into specific cells including osteoblasts (Figure 3) [22,23]. EVs from iPSCs and iPSC-derived MSCs (iMSCs) represent a novel approach for regenerative medicine [24]. Several other tissue-specific stem or progenitor cells have been investigated for their ability to generate osteoinductive EVs. These include dental pulp stem cells (DPSCs), periodontal ligament stem cells (PDLSCs), and synovial mesenchymal stem cells (SYN-MSCs) [25,26,27,28,29,30,31,32,33].

Canonical EV markers, including CD9, CD63, CD81, TSG101, and Alix, as well as MSC-specific markers such as CD73, CD90, and CD105, help define the MSC-EVs population [34]. EVs typically carry bioactive cargo, including growth factors (e.g., VEGF, HGF), cytokines, and regulatory miRNAs, which collectively contribute to their anti-apoptotic, immunomodulatory, and tissue-reparative functions [35]. Despite sharing core markers, MSC-EVs display tissue-specific molecular profiles. Proteomic and transcriptomic analyses have revealed distinct proteins and miRNAs among MSC-EVs from different sources. In particular, their specific RNA, protein, and lipid cargo can vary markedly, resulting in the differential modulation of major pathways implicated in bone formation, including Wnt/β-catenin, BMP/Smad, MAPK and Hippo signaling [19,36]. These source-dependent features also influence their behavior in vivo, where EVs demonstrate variable effects on bone repair depending on the injury model and delivery strategy. To provide a clearer and more systematic overview of these differences, Table 1 and Table 2 summarize the principal EV markers, key bioactive cargo, major signaling pathways engaged, commonly used in vivo models, and the main advantages and limitations associated with each stem cell source.

## 4. Strategies to Enhance the Osteogenic Potential of MSC-Derived Extracellular Vesicles

An increasing number of studies have explored how conditioning strategies can enhance the regenerative properties of EVs. Approaches such as the osteogenic differentiation of donor cells, exposure to hypoxia, mechanical stimulation, magnesium-based preconditioning, or genetic modification (e.g., HIF-1α overexpression) have been shown to reshape EV cargo and potentiate their pro-osteogenic effects (Table 3). Moreover, the incorporation of EVs into biomaterial-based delivery systems further improves their stability and therapeutic efficacy (Table 3). Pre-differentiated EVs exhibit superior osteoinductive potential, promoting mineral deposition and upregulating osteogenic markers when compared to EVs from undifferentiated cells [64,65,66,67,73,74,75]. These effects are mediated by the activation of signaling cascades including AMPK–mTOR, Wnt/β-catenin, BMP/Smad, and TGFβ1/Smads, with miRNAs like miR-27a-5p acting as key regulators [66,67,73]. The degree and duration of differentiation also modulate EV potency, as longer induction periods (7–14 days) yield vesicles with higher osteogenic activity [64,75]. Moreover, EV subsets demonstrate bidirectional regulatory effects, promoting both osteogenesis and osteoclastogenesis via the circ_0000722/NF-κB/AKT axis [74], suggesting a potential role in balanced bone remodeling. Despite these promising outcomes, challenges remain regarding biosafety and controlled delivery, particularly due to the use of dexamethasone-based differentiation protocols that may impact the long-term epigenetic stability [76].

It has been shown that EVs derived from mechanically stimulated BM-MSCs enhance osteoblast proliferation and differentiation by activating the Wnt/β-catenin signaling pathway and its downstream transcription factor TCF7, thus representing a promising therapeutic approach for glucocorticoid-induced osteoporosis (GIOP) [77].

A cation-activation strategy has been reported as a novel approach to modulate MSC-EVs cargo and enhance their regenerative potential [70]. In particular, EVs from magnesium-activated dental pulp stem cells (Mg^2+^-EVs) showed enhanced pro-angiogenic and pro-osteogenic activity, promoting endothelial migration, angiogenesis, BM-MSC proliferation, and osteogenic differentiation. These effects are driven largely by miR-451a-mediated activation of the AKT/eNOS pathway. Additionally, incorporating Mg^2+^-EVs into β-TCP-modified GelMA scaffolds enabled sustained release and improved bioavailability, resulting in markedly enhanced vascularized bone regeneration in a rat cranial defect model [70]. Moreover, EVs derived from magnesium-preconditioned BM-MSCs have been investigated for their therapeutic potential too. In vitro studies showed that these EVs effectively mitigated dexamethasone-induced impairment of both angiogenic function in human umbilical vein endothelial cells (HUVECs) and osteogenic differentiation in BMSCs [78]. Magnetic Ion Channel Activation (MICA) uses remote magnetic fields to stimulate mechanosensitive ion channels via magnetic nanoparticles (MNPs) and has been explored as a strategy to enhance both the production and therapeutic efficacy of EVs for bone regeneration [79]. Notably, it has been shown that MICA markedly increases EV yield from MC3T3 pre-osteoblasts compared to magnetic stimulation or TREK1(Two-Pore-Domain Weak Inward Rectifying K^+^ channel 1)-functionalized graphene oxide-MNPs alone. The EVs produced exhibited typical size, morphology, and protein markers consistent with nano-sized vesicles. Furthermore, treatment with MICA/TREK EVs significantly promoted the osteogenic differentiation and mineralization of BM-MSCs compared with EVs derived from MICA, TREK, or untreated controls [79]. The age of the donor also influences the therapeutic effects, with EVs from younger donors exhibiting enhanced regenerative potential via miR-142-5p-mediated mechanisms [72].

Similarly, hypoxic preconditioning enriches EVs with angiogenic and osteogenic factors, improving their performance in poorly vascularized environments. EV production is highly sensitive to oxygen tension, and hypoxia modulates EV composition and therapeutic efficacy [76]. EVs derived from dental stem cells cultured under oxygen restriction exhibit stronger angiogenic and immunomodulatory capacities [80]. Gao et al. demonstrated that EVs from hypoxia-preconditioned stem cells from human exfoliated deciduous teeth (SHEDs), incorporated into injectable porous PLGA microspheres with a polydopamine coating, achieved sustained release and significantly improved cranial bone regeneration [81]. Hypoxic culture highlights HIF-1α as a key regulator of adaptation [82], and genetic tools have been used to directly modulate its levels. For instance, Hernán González-King et al. showed that HIF-1α overexpression in dental pulp stem cells produced EVs enriched in Jagged1 with markedly improved angiogenic activity [83]. Marta Gómez-Ferrer et al. further combined HIF-1α and telomerase overexpression with pro-inflammatory stimulation to generate EVs with higher yield, better uniformity, and enhanced immunomodulatory effects [84].

**Table 3:** Conditioning strategies and engineering approaches. ↑ and ↓ indicate upregulation and downregulation, respectively.

Strategy	Stem Cell Type/Condition	Main Modifications in EV Cargo	Functional Effects on Osteogenesis/Regeneration	Biomaterial Delivery (If Present)	References
Osteogenic/odontogenic induction	Dental pulp stem cells (DPSCs), SHEDs, PDLSCs under osteogenic or odontogenic induction	↑ Pro-osteogenic miRNAs (e.g., miR-27a-5p), changes in AMPK–mTOR, Wnt/β-catenin, BMP/Smad, TGFβ1/Smads signaling	↑ Mineralization; ↑ RUNX2, ALP, BMP2, OCN; enhanced osteoinduction vs. naïve EVs; effects depend on induction duration (7–14 days)		[64,65,66]
Bidirectional EV subsets	Dental stem cell EV subpopulations	circ_0000722 enrichment NF-κB/AKT regulation	Dual action: promotes osteogenesis and osteoclastogenesis; implications for bone remodeling		[74]
Mechanical stimulation	Mechanically stimulated BMSCs	Modulation of Wnt/β-catenin pathway	↑ Osteoblast proliferation and differentiation; promising for GIOP		[77]
Magnesium preconditioning (Mg^2+^ activation)	Mg^2+^-activated DPSCs → Mg^2+^-EVs	↑ miR-451a AKT/eNOS activation	↑ Endothelial migration, angiogenesis, BMSC proliferation and osteogenesis; enhanced vascularized bone regeneration	β-TCP-modified GelMA scaffold (sustained release)	[70]
Magnesium-preconditioned BMSCs	BMSCs treated with Mg^2+^	Modulation of angiogenic and osteogenic cargo	Rescue of Dex-induced impairment in HUVEC angiogenesis and BMSC osteogenesis		[78]
MICA (Magnetic Ion Channel Activation)	MC3T3 pre-osteoblasts exposed to MICA + TREK1-functionalized nanoparticles	Increased EV output; preserved EV markers and morphology	↑ Osteogenic differentiation and mineralization in BMSCs vs. controls		[79]
Donor-age–dependent effects	EVs from young donors	↑ miR-142-5p	↑ Osteogenesis and bone homeostasis		[72]
Hypoxia preconditioning	Dental stem cells under low O_2_ (1–5%)	Enrichment in angiogenic/immunomodulatory factors	↑ Angiogenesis and immune modulation; enhanced therapeutic profile		[80]
Hypoxia + biomaterial delivery	Hypoxia-preconditioned SHEDs	Cargo unchanged in morphology but ↑ osteogenic and angiogenic potential	Significantly improved cranial bone regeneration	Injectable porous PLGA microspheres with polydopamine coating	[81]
Genetic modification: HIF-1α overexpression	DPSCs overexpressing HIF-1α	↑ Jagged1	Markedly enhanced angiogenesis; potential for ischemic disorders		[83]
Genetic modification: HIF-1α + telomerase + inflammatory priming	Engineered MSCs (HIF-1α + TERT + cytokine stimulation)	↑ EV yield and uniformity; enhanced immunomodulatory profile	Stronger regenerative and immune-modulating functions		[84]
General note on hypoxia sensitivity	Multiple MSC sources	Hypoxia modifies EV cargo through regulation of oxygen-sensitive pathways	Improved performance in avascular or load-bearing defects		[76,85]
General genetic engineering approaches	MSCs engineered for osteoinductive genes/miRNAs	Customized EV cargo	Targeted functional enhancement for bone repair		[86]

## 5. In Vitro and in Vivo Models of EV-Mediated Bone Regeneration

In recent years, both in vitro and in vivo models employing EVs have been extensively investigated for their potential in bone regeneration (Table 4). As therapeutic tools, EV-functionalized scaffolds have been suggested as a delivery method to improve EV retention and enhance the effectiveness of future healing [87,88]. In vivo findings demonstrated that a 3D scaffold with human gingival mesenchymal stem cells (hGMSCs)-EVs implanted into rats with damaged cortical calvaria bone tissue enhanced bone repair by exhibiting improved osteogenic activity [89]. Furthermore, the authors showed that transforming growth factor (TGFβ), bone morphogenetic proteins (BMP2), tuftelin 1 (TUFT1), and tuftelin 11 (TFIP11) were all upregulated in human periodontal-ligament stem cells (hPDLSCs) and hGMSCs. Scaffolds associated with EVs increase the capacity to commit toward the osteogenic lineage [90]. Additionally, Pizzicannella J et al. assessed the ability of a collagen membrane (3D-COL) loaded with EVs or EVs engineered with polyethylenimine (PEI-EVs) and hPDLSCs to promote bone repair in rats with calvarial defects. Consistently, in vitro data have revealed that hPDLSCs cultivated with 3D-COL and PEI-EVs express more osteogenic markers [91].

Furthermore, when combined with scaffolds composed of polylactic–co-glycolic acid and magnesium–gallic acid, ADSC-EVs enhanced angiogenesis, osteogenesis, and anti-inflammatory responses in severe bone deficiencies [92], and in a rat femoral defect model, polydopamine-coated gelatin sponge scaffolds loaded with ADSC-EVs showed a notable increase in osteogenic marker expression [93].

Moreover, in a rat model of osteochondral defects, a bilayer decellularized ECM hydrogel incorporating ADSC-EVs and produced via three-dimensional bioprinting effectively promoted the concomitant regeneration of cartilage and subchondral bone [94]. In a different study, it has been demonstrated that injectable gelatin-nanoparticle hydrogels enhanced with EVs harboring miR-451a promoted M2 macrophage polarization and bone repair in a rat calvaria lesion model [71].

An in vitro study showed that iMSC-EVs, when co-cultured with OA chondrocytes, have significant potential as a novel treatment for osteoarthritis (OA) and cartilage regeneration [95]. In addition, iMSC-EVs act as key mediators of cartilage repair by preserving cartilage homeostasis against inflammatory damage, as demonstrated by their ability to reduce TNF-α–induced collagenase activity and to counteract the TNF-α–mediated upregulation of COX-2 and pro-inflammatory interleukins [96].

Furthermore, it has been shown that UC-MSC-EVs embedded in hydrogel represent an interesting tool for promoting the healing of rat bone lesions in vivo. In fact, the histological analysis revealed that the hydrogel-EVs group had the highest microvessel density and most new bone tissue [88,97].

Additionally, an in vivo study demonstrated that mechanically activated extracellular vesicles (MA-EVs) produced from pro-osteogenic osteocytes offer a possible cell-free treatment to improve bone regeneration and repair in conditions like osteoporosis [98].

Interestingly, Du Z. et al. demonstrated that producing MSC-derived EV using BMP2 and BMP7 could effectively supply bioactive protein, and following EV uptake, recipient cell BMPRI/II and SMAD phosphorylation were activated, resulting in improved bone repair in a rat calvarial defect model [99].

Again, an in vivo investigation revealed that PDLSC-EVs immobilized in Matrigel provided a topical cell-free transplantation technique and increased cell infiltration, which accelerated the repair of bone tissue [100]. Additionally, PDLSC-EVs enhanced BM-MSCs migration and proliferation in vitro through increased phosphorylation of AKT and extracellular signal-regulated kinase 1/2 (ERK1/2), mediated by the activation of adenosine receptor signaling [100].

A different method to use EVs for bone regeneration was found by Qui et al., which modified exosomes (BT-Exo-siShn3) with a bone-targeting peptide to accurately deliver siRNA to osteoblasts. Shn3 knockdown in osteoblasts stopped the production of osteoclasts by promoting osteogenic differentiation and lowering autologous receptor activator of RANKL expression, suggesting a therapeutic tool for osteoporosis [101].

In addition, micro-computed tomography, histological, and immunohistochemical investigations showed that the MSC-EVs, in association with alginate–polycaprolactone (PCL), a biocompatible material, combined with osteodifferentiated MSCs, improved vasculature formation and tissue-engineered bone regeneration in a nude mouse subcutaneous bone formation paradigm [102].

Dynamic bioreactors and microphysiological systems (MPS) are increasingly employed to culture EVs under conditions that more closely mimic the in vivo cells’ microenvironment. A recent study demonstrated that culturing MSCs in a three-dimensional bioreactor system (VITVO^®^) significantly increases the yield and purity of MSC-EVs compared with conventional 2D cultures, highlighting the value of 3D dynamic platforms for scalable EVs production and for preserving their therapeutic potency [103]. However, although bioreactor-based systems have been successfully employed to enhance the large-scale production and bioactivity of MSC-EVs, [104,105], these platforms have not yet been specifically applied to generate or optimize EVs for bone-related applications. Microphysiological systems, such as bone-on-a-chip and microfluidic osteogenic platforms, have been successfully developed to mimic key features of bone biology, including 3D osteogenic differentiation, co-culture of osteoblasts and osteoclasts, and perfusion-based mechanical stimulation [106,107,108]. However, to date, no studies have specifically used these models to investigate EVs-mediated communication in bone. Furthermore, innovative organoid models represent a useful tool to evaluate the role of EVs in bone regeneration. Unlike simple 3D spheroids, which are typically homogeneous cell aggregates lacking spatial organization [109], or scaffold-based cultures, in which the architecture is externally imposed [110], bone organoids are generated by culturing pluripotent or tissue-derived progenitor cells that differentiate and self-organize into 3D systems. These models ideally exhibit intrinsic tissue patterning, extracellular matrix deposition, and progressive mineralization [111]. Recent reports on bone/cartilage organoid engineering confirm this conceptual framework and emphasize the importance of cell type selection, matrix composition, and induction protocols for achieving tissue-level complexity and functionality [112]. Within such organoid platforms, EVs may serve as conditioning factors that enhance osteogenic differentiation, matrix deposition, angiogenesis, or immunomodulation; as functional readouts, with organoid-derived EVs reflecting osteogenic, osteoclastic, or stromal activity and providing non-destructive biomarkers of bone formation or pathology; and as therapeutic agents in organoid-based models of bone defects or disease to assess their regenerative potential [113]. Organoid-derived extracellular vesicles (OEVs), especially those from bone organoids, hold strong therapeutic potential but remain at an early stage of development. Their nanoscale size, safety, and cell-free nature make them effective nanocarriers, with natural OEVs showing promising results and engineered OEVs offering targeted delivery and enhanced therapeutic effects [114,115]. However, key challenges such as organoid production, OEV isolation, engineering strategies, drug loading, and controlled release must still be addressed.

## 6. Clinical Perspectives on Extracellular Vesicles for Bone Regeneration

### The Regulatory Potential of Extracellular Vesicles in Osteonecrosis

Osteonecrosis represents a clinical condition in which impaired vascularity compromises bone integrity, and EVs have recently been explored as regulators of osteogenesis and microenvironmental repair. Osteonecrosis (e.g., avascular necrosis of the femoral head) is characterized by osteocyte apoptosis and bone matrix degradation due to impaired blood supply, ultimately leading to structural collapse and functional loss [116]. Recent studies have highlighted the potential of EVs in regulating osteogenesis for osteonecrosis intervention [117,118,119]. In fact, the evidence indicates that intravenous infusion of urine derived stem cells (USC)-EVs during the early phase of glucocorticoids treatment mitigates angiogenic dysfunction, limits apoptosis in trabecular and marrow cells, and preserves bone integrity and microstructural organization in the femoral heads of rats [118]. In vitro experiments further confirmed that USC-EVs alleviate GC-mediated suppression of endothelial angiogenesis and apoptotic signaling [118]. Moreover, administration of BM-MSC-EVs enriched with miR-148a-3p in rats with osteonecrosis of the femoral head enhanced cell proliferation and osteogenic activity, thereby attenuating disease progression [119]. Mechanistically, miR-148a-3p targets Smad ubiquitination regulatory factor 1 (SMURF1), an E3 ubiquitin ligase that promotes the degradation of SMAD7, a key regulator of the TGF-β signaling pathway involved in osteogenic differentiation. By inhibiting SMURF1, miR-148a-3p preserves SMAD7 levels and upregulates BCL2, an anti-apoptotic protein that supports bone marrow cell survival [119]. Collectively, the miR-148a-3p/SMURF1/SMAD7/BCL2 axis contributes to maintaining osteoblast viability and bone integrity, thereby alleviating osteonecrosis of the femoral head.

As intercellular signaling carriers, EVs transport bioactive molecules to precisely modulate target cell functions and they optimize the osteonecrotic microenvironment by inhibiting the release of inflammatory factors and reducing osteoblast apoptosis. In particular, it has been demonstrated that MSC-EVs can counteract zoledronic acid induced senescence in stem cells, osteoblasts, and fibroblasts, while concurrently reducing the expression of pro-inflammatory cytokines, including IL-6 and IL-8, as well as matrix metalloproteinases MMP-1 and MMP-3 [120]. In addition, EV-AT treatment enhanced the proliferation, migration, and tube formation capacity of HUVECs, which were otherwise suppressed by zoledronic acid [121]. Encouraging results came from HUVECs and BM-MSCs-EVs, that seem to improve the osteonecrosis of femoral head induced by glucocorticoid treatment in murine models [122,123], as well as from adipose tissue stem cells derived EVs overexpressing miR-378 [124]. Considering the impact that osteonecrosis of the femoral head can have on daily living activities and the scarce treatment options, these clinical EVs applications should be better investigated in human models [125]. Overall, EV-based approaches in osteonecrosis show promising translational potential by enhancing osteoblast survival, angiogenesis, and microenvironmental stability. However, the clinical application remains limited by the need for optimized delivery systems, dosing strategies, and safety validation.

## 7. Extracellular Vesicles in Osteoporosis: Possible Therapeutic Targets

Beyond osteonecrosis, EVs have also been explored for osteoporosis-related bone loss. Osteoporosis is characterized by reduced bone mass and impaired remodeling, and EVs are increasingly investigated as modulators of osteoblast–osteoclast balance [126]. Data from clinical studies have displayed that serum EVs miRNAs are differentially expressed in postmenopausal osteoporosis. In particular, miR-3-766p and miR-3-1247p were found to be associated with reduced bone mineral density [127]. Likewise, exosomal circFAM63B suppresses bone regeneration, and may be a target for anti-osteoporosis therapies as with the aforementioned microRNAs [128].

On the other hand, in an ovariectomy-induced postmenopausal osteoporosis mouse model, osteoblastic differentiation was induced with EVs derived from bone marrow stromal cells and then conjugated with a BM-MSC-specific aptamer. Hence, aptamer-conjugated EVs may be another therapeutic strategy for osteoporosis [129]. Promising results were also detected when EVs were obtained from overexpressing glycoprotein non-melanoma clone B MSCs, with in vivo data showing improved trabecular bone regeneration and mitigation of the osteoporotic phenotype [130]. The Wnt/β-catenin pathway seems to be activated by BMSCs EVs, with attenuation of trabecular bone loss [131]. Furthermore, extracellular vesicles derived from UC-MSCs had positive effects on the bone regeneration of a metaphyseal femoral defect in osteoporosis rat models [132].

Interestingly, EV treatments appear to have low systemic toxicity [133]: considering these results, future research should deepen the possible therapeutic roles of tailored EVs, including apoptotic bodies, in humans [134,135].

Clinical conditions that can negatively impact quality of life in osteoporosis are pathological vertebral fractures, which can be also caused by inadequate blood supply. Some remarkable results were obtained with an injectable silk gel scaffold that could promote bone healing by gradually releasing MSCs-EVs and HIF-1α pathway activator [136]. BM-MSCs-EVs combined with a nanocement carrying BMP-2 and zoledronate exhibited positive effects on femoral neck osteoporosis, with possible perspectives in this detrimental clinical setting [137].

However, some conflicting results emerged when the anti-osteoclastogenic activity of EVs was studied in preventing autograft resorption in vertebral fractures, compared to perivascular stem cells activity [138]. Some authors contrarily found that PKH-26-labeled MSCs and osteoblasts EVs implanted with hydroxyapatite nanocement regenerated tibial bone defect in murine models, while others specifically highlighted the role of matrix vesicles [139,140].

Thus, these findings suggest that an appropriate nanocement or hydrogel, may enhance the effectiveness of EVs-mediated bone regeneration and healing, with important clinical implications [141].

On the other hand, the optimal biomechanical load can prevent pathological bone fractures; in this field, the key role of EVs was demonstrated. In fact, EVs can physiologically recruit MSCs, and, by paracrine effects, converge them to osteogenic commitment [98]. Analogously, trichostatin A EVs administration replicated exercise-induced effects on the bone architecture and bone mass of osteoporotic mice [142].

Other studies showed that only early passage human MSCs-conditioned medium reversed osteoporotic alterations, with anti-senescence effects in bone marrow MSCs, while bone MSCs-EVs preconditioned with Epimedium (a plant-derived element) could stimulate osteogenesis by targeting autophagy [143,144]. Research focused on other plant-based (i.e., Aesculetin, Pueraria Lobata) derived EVs showed effects on osteoporosis, promoting the differentiation of human bone marrow MSCs and enhancing autophagy in vitro and in ovariectomized rats [145,146]. Similar results were described for yam-derived EV-like nanovesicles, by promoting the mineralization of osteoblasts, even when orally administered [147]. Recent research also showed that bovine milk contains EVs that can enhance human osteogenic Saos-2 cells and osteogenic gene GJA1, as well as promoting bone mineral density of tibia in murine models [148,149]. Taken together, these findings suggest that a nutraceutical approach combined with EV interventions may serve as an effective adjunct therapy for osteoporosis.

On the other hand, EVs can reverse drug induced bone loss, such as in valproic acid-induced bone mineral density decrease and in glucocorticoid induced osteoporosis [150]. In fact, targeting osteoclast precursors with anti-microRNA-6359 EVs and positively modulating osteogenesis/angiogenesis, respectively, seem to be protective against these conditions [151]. These findings support the translational relevance of EVs as biomarkers and therapeutic agents in osteoporosis. Still, the heterogeneity of EV preparations, short in vivo half-life, and limited clinical trials remain important challenges for therapeutic implementation.

## 8. Bone Defect Repair and Fracture Healing: The Importance of Scaffolds and Microenvironment

In addition to systemic metabolic bone disease such as osteoporosis, EVs play a role in localized bone defects. Critical-sized bone defects and fracture non-unions require regenerative strategies that support osteogenesis, angiogenesis, and inflammation control. EVs are emerging as potent adjuncts to biomaterial-based repair. Bone defects are a challenging clinical condition that require orthopedic treatment, including bone grafts and induced membrane techniques [152]. 

Large bone defects can also be treated with distraction osteogenesis, which is a surgical technique that acts by creating an osteotomy and then gradually separating the bone segments, which stimulates the body to create new bone to fill the gap [126]. In this field, MSCs-EVs can promote new bone formation and repair in rat models [73,153,154]. Similarly, the paracrine effects of exosomes and conditioned medium from sinus mucosa-derived cells and periosteum-derived cells increased osteogenesis both in vitro and in rat models of bone femoral defect [155]. EVs derived from mature dendritic cells displayed similar results in a bone femoral defect [156]. Interestingly, inflammation in these condition may have deleterious effects on healing; in fact, polarization of macrophages can change the EVs cargo and subsequently bone regeneration [157]. Based on these findings, the combination of anti-inflammatory therapy with EVs-based treatment warrants investigation in future studies.

Stimulation of skeletal cell proliferation, migration, and osteo-differentiation could also result from perivascular stem cells’ (PSCs) activity, with intriguing possibilities for a future therapeutic approach [158].

However, the main obstacle in obtaining bone repair is targeting a “suffering” bone that exhibits prolonged oxidative stress and less active MSCs. To overcome this issue (i.e., bypassing lysosomal degradation), several studies have enriched EVs using bioengineering strategies, such as incorporating iron–tannic acid complexes [159].

Other authors integrated MSCs-EVs with polydopamine and delivered them in PLGA porous microscaffolds, which exhibited high load efficiency and long-term efficacy in human deciduous teeth and in rat cranial bone models [160]. 

On the other hand, hydrogel microparticles can support the long-term effects of EVs obtained from bone marrow MSCs during fracture healing by coupling osteogenesis and angiogenesis [161]. Similarly, EVs derived from M2 macrophages can attenuate inflammation and promote osteosynthesis in non-healing bone fractures when combined with a specific BM-MSCs aptamer [162]. Taken together, these studies underline the importance of different EV types in acute and non-healing fractures [163,164,165]. The use of EV-loaded scaffolds and engineered EVs shows promising translational potential for large bone defect repair. However, clinical translation depends on standardizing EV isolation, scaling production, and ensuring targeted delivery in compromised bone tissue.

## 9. Diabetic Bone-Specific Physiopathology May Affect Therapeutical Results

While these strategies highlight the regenerative potential of EV-based approaches for bone repair, their effectiveness can be profoundly influenced by specific pathological conditions. Among these, diabetes represents a major factor capable of altering bone physiology and modulating the therapeutic response to EVs. Type 2 diabetes and hyperglycemia impair the reparation process of bone tissue, triggering the so-called diabetic bone osteopathy. Engineered EVs—acting on endoplasmic reticulum regulation, ZEB1 and possibly on Wnt/β-catenin and PI3K/AKT pathways—effectively enhanced osteogenesis and neoangiogenesis in diabetic osteopathy [166,167,168]. On the other hand, in vitro and in vivo data show that a hyperglycemic setting can reduce the effectiveness of BMSCs and macrophage EVs, thus highlighting the importance of blood glucose control for bone healing in diabetes [169,170].

## 10. Extracellular Vesicles in Enthesis Acute and Chronic Pathology

Enthesis injuries require coordinated bone–tendon regeneration, and EVs have shown immunomodulatory and osteogenic effects that may improve surgical outcomes. EVs may have effects on physio-pathological ossification in tendon-to-bone (enthesis) architecture [171]. In fact, some research highlighted the positive effects of a macroporous hydrogel complex conjugated with ADSCs-EVs after rotator cuff tendon repair. This treatment showed biomechanical improvements vs. the control group, with possible immunomodulatory effects enthesis [172]. The healing of the enthesis is of particular interest: in fact, this process can affect orthopedic surgery outcomes. A purified EV product injected after surgical repair in preclinical models promoted the confluence of osteoblasts and tenocytes [173].

In a chronic rotator cuff tear rat model, local injection of EVs-loaded sodium alginate hydrogel showed interesting results in enthesis regeneration [174]. Remarkably, in other animal models, a local application of HUVECs-EVs promoted enthesis healing even in acute rotator cuff injury [175]. EVs have the potential of becoming one of the novel therapeutic modalities in regenerative sport medicine, also showing promising results in soft tissue healing [176]. Although EV-based therapies enhance biomechanical recovery in preclinical models, optimized dosing, delivery platforms, and long-term safety remain barriers to clinical use.

## 11. Osteoarthritis and Related Clinical Complications—The Role of Subchondral Bone

OA progression involves chronic inflammation and subchondral bone remodeling, making EVs attractive candidates for cartilage and bone regeneration. MSC injections are currently used as a treatment for osteoarthritis (OA); however, growing evidence indicates that MSC-EVs can also promote cartilage regeneration and subchondral bone repair, as demonstrated by histomorphometric analyses [177]. In fact, MSC-EVs from different tissues (bone marrow, adipose tissue and umbilical cord) showed anti-inflammatory properties in vitro and in ex vivo models; BM-MSCs-EVs demonstrated a superior therapeutic effect [178]. However, other authors reported that EVs obtained from MSCs of adipose tissue may be more effective for cartilage and bone regeneration [1]. Similar results in mouse models with induced OA were obtained with platelet-derived EVs: interestingly, subchondral bone abnormal remodeling was reduced [179]. This outcome is of particular interest in clinical practice, since the degeneration of subchondral bone affects all the articular biomechanical properties. Remarkably, EVs from subchondral bone MSCs are key regulators of the cross-talk with altered cartilage [58]. Targeting this mechanism could have a crucial role for the prevention and treatment of OA.

In this setting, a hydrogel scaffold with 3D printing was effective in ameliorating the effects of EVs on subchondral bone in preclinical OA rat models [94]. In murine OA models, scaffolds with decellularized extracellular matrix could also improve articular cartilage repair [180]. The appropriate hydrogel seems to be pivotal in achieving articular benefits even in platelet rich plasma therapies [181]. Some promising therapeutic results were also seen in osteochondral injury models, in which human Wharton’s jelly and embryonic MSCs-EVs respectively reduced inflammatory pathways and regulated the BMP2/RUNX2 axis with histological results [182,183].

On the other hand, EVs released by osteoblasts from patients with coxarthrosis exerted negative effects on cellular metabolism and impaired the osteogenic differentiation of MSCs [184]. The same findings were present when osteoporotic bone-derived osteoblasts EVs were evaluated [184]. Therefore, the physiological state of EV-producing cells must be carefully considered when developing EV-based regenerative strategies in clinical practice, as EVs derived from diseased tissues may counteract therapeutic goals [184].

Noteworthy data are rising from human-urine derived stem cells (hUSCs) EVs and knee osteoarthritis: miR-140s transfected hUSCs EVs may be a possible treatment for the progression of subchondral bone alterations when injected into the affected joint [185].

Clinical complications of OA can necessitate total joint replacement. In this scenario, EVs from hUSCs appear to mitigate osteolysis mediated by plastic prothesis materials in murine models [186]; other EVs and scaffolds showed interesting results, although data on human models are lacking [187,188]. Thus, EVs show strong potential to modulate inflammation and restore osteochondral homeostasis; however, clinical translation will require scalable production and effective integration with biomaterial carriers. 

## 12. Maxillo-Facial Defects, Periodontal Pathologies, and Dental Implant Issues

Craniofacial defects, periodontal disease, and implant-related bone loss represent conditions where localized EV therapies may offer targeted regeneration. Recently it has been reported that MSCs-EVs, irrespective of their specific population, can improve the osteogenic repair response in maxillary bone or periodontal defects [189]. On the other hand, orofacial MSCs-EVs dysregulation seems pivotal in the development of orofacial deformities, suggesting that the GATA4-miR-206-3p axis may be a target for future therapies of these impactful diseases [190].

Guided bone regeneration is commonly used for alveolar bone augmentation. In particular, gingival and dental follicle MSCs-EVs have great potential in maxillary bone tissue regeneration [191]. Interestingly, EVs derived from gingival SCs enhanced the migration and osteogenic differentiation of pre-osteoblasts [192]. These results could also have clinical insights for oral implants, as suggested by Wang et al., who demonstrated that osteoblasts EVs containing ALKBH5 may mitigate peri-implant bone resorption [193]. Furthermore, immunomodulatory effects of MSCs EVs, enhancing osseointegration of dental implants, were described in bone defects and diabetic osteopathy [194,195].

Similarly, promising results for translational applications were obtained with human dental pulp stem cells (hDPSCs)-derived EVs, promoting osteogenic differentiation in hADSCs for maxillofacial bone regeneration [28]. Analogous results were obtained by using BM-MSC-EVs on periodontal regeneration [196]. However, caution is required for clinical application, as these effects depend on the specific microenvironment of maxillofacial tissues [197]. Future research should focus on modulating EVs secretion, which may represent a strategy to enhance periodontal bone regeneration under inflammatory conditions [198].

On the other hand, EVs were dysregulated in ameloblastoma and odontogenic keratocyst, suggesting possible therapeutic strategies in benign odontogenic lesions [199]. Radiation therapy, which is commonly used for head and neck tumors, can alter the bone architecture and the miRNA cargo of plasma exosomes. These alterations could affect the Wnt and MAPK signaling. In fact, EVs were suggested to act as mediators of radiation-induced bone injury and possible targets for therapy [200].

The potential therapeutic effects of EVs have been described in preventing senescence of adipose tissue stem cells, osteoblasts, and fibroblasts in models of bisphosphonate-related osteonecrosis of the jaw [120,121]. In rats with zoledronic acid-related osteonecrosis of the jaw (BRONJ)-like lesions, the administration of ADSCs–EV significantly ameliorated the BRONJ-like pathology by promoting soft tissue regeneration and improving overall bone healing [121]. Similarly, EVs may help in healing large jawbone defects with some data on macrophage-derived EVs in this preclinical setting [201,202].

Additionally, EVs also showed interesting results in periodontitis for counteracting anaerobic pathogens’ replication [203]. Some remarkable data have emerged on aspirin-loaded EVs and on three-dimensional microenvironment-cultured EVs in alveolar bone defects [204,205,206]. Thus, MSCs-EVs seem to be a promising treatment for inflammatory bone loss in periodontitis animal models, although future research is needed in humans [207].

## 13. Opportunities and Limitations of AI/ML in EV-Mediated Bone Repair

Despite the rapid progress in the EV field, major obstacles remain in achieving reproducible protocols for isolation, quantification, and potency assessment. In addition, GMP-compatible large-scale manufacturing is still technically challenging. Recent advances in closed and automated bioreactor systems, together with a technique called tangential flow filtration (TFF), have demonstrated promising improvements in EV yield, purity, and batch-to-batch consistency, representing concrete steps toward scalable production, although their clinical implementation is still at an early stage [105,208,209,210]. At the research level, multi-omics technologies, including proteomics, transcriptomics, metabolomics, and lipidomics are increasingly being employed to dissect EV heterogeneity and identify molecular signatures associated with regenerative potency, such as miRNA profiles or pro-osteogenic and pro-angiogenic proteins [211,212,213,214]. Moreover, artificial intelligence (AI) is increasingly emerging as a powerful tool for EV analysis and development: for example, deep-learning has been used to segment small extracellular vesicles in TEM images [215], while machine-learning models have been proposed to classify EV subtypes from spectral or other high-dimensional data and to support EV-based diagnostics and potentially functional prediction [216,217]. However, although artificial intelligence and machine learning (AI/ML) are increasingly being applied to extracellular vesicle (EV) research, mainly for subtype classification, cargo analysis, and multi-omics integration, no studies have yet used ML to predict or enhance the osteogenic potential of EVs. Therefore, despite its clear potential for improving EV standardization and functional assessment, the use of AI/ML in EV-based bone regeneration remains at a conceptual and exploratory stage.

## 14. Challenges for Clinical Translation of EV-Based Bone Regeneration

Despite promising preclinical results across osteonecrosis, osteoporosis, critical-sized bone defects and fracture non-unions, osteoarthritis and subchondral bone pathology, maxillofacial and periodontal conditions, diabetic bone disease, and enthesis injuries, several hurdles still limit the clinical translation of EV-based therapies for bone regeneration. Most available data derive from small animal models using heterogeneous EV sources, isolation protocols, and dosing regimens, leading to a marked variability in EV preparations and making it difficult to define standardized GMP-compatible production workflows and robust quality criteria, as highlighted by the divergent results observed across osteoporosis and fracture models [138,139,140,143,144,145,146,147].

The EV cargo composition is strongly influenced by the originating cell type, passage, microenvironment, and disease state, as shown for osteoporotic and coxarthrotic bone–derived osteoblast EVs, which impaired MSC osteogenic differentiation [184], raising concerns about reproducibility, potential immunogenicity, and the risk that EVs from pathological tissues may counteract regenerative goals [184].

In addition, the short in vivo half-life of EVs and the need to target “suffering” bone characterized by oxidative stress and impaired MSC activity have prompted the development of complex delivery systems—including hydrogels, nanocements, and porous microscaffolds—which enhanced regeneration in models of osteoporosis, bone defects, and jawbone pathology [136,137,138,160,161,204,205,206] but also introduced challenges for large-scale manufacturing, storage, and regulatory classification.

Recent advances in closed bioreactor systems and tangential flow filtration (TFF) have shown improved EV yield and batch-to-batch consistency [105,208,209,210], while multi-omics profiling has helped characterize EV heterogeneity and identify regenerative cargo signatures [211,212,213,214]. Furthermore, AI/ML approaches are emerging for EV subtype identification and cargo analysis [215,216,217]. However, these strategies remain at an early exploratory stage, and no studies have yet applied ML to predict or enhance the osteogenic potential of EVs, indicating that significant barriers remain before standardized clinically applicable EV-based bone regenerative products can be realized.

## 15. Conclusions

Extracellular vesicles derived from various stem cell sources represent a promising tool for promoting bone regeneration due to their ability to modulate osteogenic differentiation, angiogenesis, and immune responses. Differences in EV source, conditioning strategies, and in vitro models significantly influence experimental outcomes, underscoring the need for standardized protocols to improve reproducibility and comparability. Thus, EVs hold transformative potential for osteogenic therapies. Addressing current challenges through interdisciplinary efforts in bioengineering, standardization, and preclinical modeling will be critical to unlocking their clinical promise. Recent advances in the bioengineering of extracellular vesicle (EV) cargo and surface characteristics, along with the introduction of innovative three-dimensional (3D) culture platforms and emerging organoid-inspired approaches for bone research, have improved our understanding of EV biology and broadened the potential therapeutic applications, despite the fact that bona fide bone organoids remain in their early developmental stage. Ultimately, overcoming current challenges in scalable production, quality control, and regulatory approval will be essential for translating EV-mediated osteogenesis into effective clinical therapies.

## Figures and Tables

**Table 1:** Stem cell sources of EVs.

Stem Cell Source	Typical EV Markers	Key Cargo	Main Signaling Pathways, Protein Modulation	Main In Vivo Models	Advantages/Limitations	References
BM-MSCs	CD9, CD63, CD81, TSG101	miR-186, miR-196a, miR-27a, miR-150-3p, miR-424-5p, combined let-7a-5p, let-7c-5p, miR-328a-5p, and miR-31a-5p proteins ((Fibulin-1 (FBLN1), Prolargin (PRELP), Matrix Gla protein (MGP), Cystatin-C (CST3), SCUBE3, FGFR1, CCN5, IGFBP4, CTHRC1, IGFBP6, Decorin (DCN), Tetranectin (CLEC3B), MMP2, IGF2, IGFBP2, IGFBP3, HMGB1, COL6A1, Versican (VCAN)	Wnt/β-catenin pathway Hippo pathway BMP/Smad pathway	Calvarial defect, osteoporosis	Highly osteogenic; invasive sourcing	[14,19,36,37,38,39,40,41,42,43,44]
ADSCs	CD9, CD63, CD81	miR-375, autophagy regulators	MAPK pathway, Ras protein activity	Bone defect repair	High yield; variability among donors	[20,42,45,46]
UC-MSCs	CD9, CD63, CD81	CLEC11A, miRNAs	RUNX2 HIF-1α/VEGF	Fracture healing	Low immunogenicity; perinatal source	[21,47,48,49]
iMSCs	CD9, CD63, CD81	miR-196a, miR-206, Wnt proteins	Wnt/β-catenin pathway	Calvarial defect, angiogenesis	Scalable; requires reprogramming	[50,51,52,53]
DPSCs	CD9, CD63, CD81, TSG101	miR-27a, RUNX3, Rab27a	Wnt/β-catenin pathway MAPK pathway	Bone and periodontal defect	Minimally invasive; tooth source	[28,29,54,55]
SYN-MSCs	CD9, CD63, TSG101	chondrogenic/pro-repair miRNAs and proteins (Sox9-related)	PI3K/Akt pathway MAPK pathway	osteochondral repair, osteonecrosis of femoral head (ONFH) models	cartilage-specific regenerative potential; scalable production using 3D bioreactors; donor/inflammation status affects yield	[25,33,56,57,58,59]

**Table 2:** Pro-osteogenic and anti-osteogenic miRNAs across EV sources.

miRNA	EV Source(s)	Functional Role	Key Pathways/Targets	Effect on Osteogenesis	Refs
miR-186	BM-MSC-EVs	Pro-osteogenic	Mob1/Hippo inhibition	↑ Osteoblast proliferation	[19]
miR-150-3p	BM-MSC-EVs	Pro-osteogenic	↑ Runx2, Osterix; ↓ apoptosis	↑ Osteogenesis	[36]
miR-935	BM-MSC-EVs	Pro-osteogenic	STAT1 inhibition → ↑ Runx2	↑ Osteogenic differentiation	[60,61]
miR-196a	BM-MSC-EVs; iMSC-EVs	Pro-osteogenic	Targets Dkk1 → Wnt/β-catenin activation	↑ ALP, OCN, Runx2	[14,62,63]
miR-27a	BM-MSC-EVs; iMSC-EVs	Pro-osteogenic	Targets Dkk2	Protects against bone loss; ↓ osteoclasts	[14,37,38,63]
miR-27a-5p	Dental stem cell EVs	Pro-osteogenic	AMPK–mTOR, Wnt, BMP/Smad	↑ Mineralization	[64,65,66,67]
let-7a-5p/let-7c-5p/miR-328a-5p/miR-31a-5p	BM-MSC-EVs	Pro-osteogenic	BMP/Smad modulation	↑ Smad1/5/9; ↓ Smad2/3	[43]
miR-21	OP-BM-MSC-EVs	Anti-osteogenic	Targets Smad7	↓ ALP, Runx2	[68]
miR-424-5p	OP-BM-MSC-EVs	Anti-osteogenic	↓ WIF1 → Wnt disruption	↓ Osteogenic differentiation	[39]
miR-375	ADSC-EVs	Pro-osteogenic	Targets IGFBP3	↑ Bone regeneration	[45]
miR-1263	UC-MSC-EVs	Pro-osteogenic	Mob1/Hippo	↓ Apoptosis; ↑ balance osteoblast/adipocyte	[47,69]
miR-451a	Mg^2+^-EVs; hydrogels	Pro-osteogenic	AKT/eNOS activation	↑ Angiogenesis and osteogenesis	[70,71]
miR-142-5p	Young-donor EVs; PDLSC-EVs	Pro-osteogenic	Age-dependent enrichment	↑ Osteogenesis	[64,72]
miR-122-5p/miR-25-3p/miR-192-5p	PDLSC-EVs	Pro-osteogenic	Osteogenic pathway regulators	↑ Osteogenic differentiation	[64]
miR-206	BM-MSC-EVs; iMSC-EVs	Pro-osteogenic-related	Enriched osteogenic miRNA	Supports osteogenic gene upregulation	[14,63]

**Table 4:** Summary of in vitro and in vivo models using EVs: sources, delivery strategies, and key outcomes.

EV Origin	Model and Delivery	Key Outcomes	Refs
Periodontal MSC-derived EVs (hGMSC/hPDLSC)	In vitro and rat calvarial models; 3D scaffolds/collagen membranes	Enhanced osteogenesis, signaling upregulation (TGFβ, BMP2), improved bone repair	[87,88,89,90,91]
ADSC-EVs (incl. engineered and 3D-bioprinted systems)	Rat femoral and osteochondral defects; PLGA/Mg-GA, polydopamine, ECM hydrogels	Improved angiogenesis, osteogenesis, reduced inflammation; cartilage–bone dual regeneration	[71,92,93,94]
Induced MSC-EVs and hucMSC-EVs	In vitro OA chondrocytes; rat bone defect w/hydrogel delivery	Protection of cartilage homeostasis; increased vascularization and bone formation	[88,95,96,97]
Osteocyte-derived mechanically activated EVs (MA-EVs)	In vivo osteoporotic models; local delivery	Promoted bone regeneration in osteoporotic conditions	[98]
BMP2/BMP7-primed MSC-EVs	Rat calvarial defect; local delivery	Activated BMPRI/II–SMAD pathway; improved bone repair	[99]
PDLSC-EVs (Matrigel) and engineered bone-targeted EVs (BT-Exo-siShn3)	Topical gel delivery; systemic/local targeted EVs	Enhanced MSC recruitment, AKT/ERK activation; Shn3 silencing → ↑ osteogenesis, ↓ RANKL	[100,101]
MSC-EVs + Alginate-PCL scaffolds	In vivo subcutaneous bone formation model	Improved vascularization and engineered bone formation	[102]

## Data Availability

Not applicable.

## References

[1] Li Q., Yu H., Sun M., Yang P., Hu X., Ao Y., Cheng J. (2021). The tissue origin effect of extracellular vesicles on cartilage and bone regeneration. Acta Biomater..

[2] Gurunathan S., Kang M.-H., Qasim M., Khan K., Kim J.-H. (2021). Biogenesis, membrane trafficking, functions, and next generation nanotherapeutics medicine of extracellular vesicles. Int. J. Nanomed..

[3] Wang B.Z., Luo L.J., Vunjak-Novakovic G. (2022). RNA and Protein Delivery By Cell-Secreted and Bioengineered Extracellular Vesicles. Adv. Healthc. Mater..

[4] Théry C., Witwer K.W., Aikawa E., Alcaraz M.J., Anderson J.D., Andriantsitohaina R., Antoniou A., Arab T., Archer F., Atkin-Smith G.K. (2018). Minimal information for studies of extracellular vesicles 2018 (MISEV2018): A position statement of the International Society for Extracellular Vesicles and update of the MISEV2014 guidelines. J. Extracell. Vesicles.

[5] Tamkovich S.N., Tutanov O.S., Laktionov P.P. (2016). Exosomes: Generation, structure, transport, biological activity, and diagnostic application. Biochem. Suppl. Ser. A Membr. Cell Biol..

[6] Krylova S.V., Feng D. (2023). The machinery of exosomes: Biogenesis, release, and uptake. Int. J. Mol. Sci..

[7] Van Niel G., D’Angelo G., Raposo G. (2018). Shedding light on the cell biology of extracellular vesicles. Nat. Rev. Mol. Cell Biol..

[8] Caruso S., Poon I.K. (2018). Apoptotic cell-derived extracellular vesicles: More than just debris. Front. Immunol..

[9] Wang Y., Wen J., Lu T., Han W., Jiao K., Li H. (2024). Mesenchymal stem cell-derived extracellular vesicles in bone-related diseases: Intercellular communication messengers and therapeutic engineering protagonists. Int. J. Nanomed..

[10] Hertel F.C., Silva A.S.d., Sabino A.d.P., Valente F.L., Reis E.C.C. (2022). Preconditioning methods to improve mesenchymal stromal cell-derived extracellular vesicles in bone regeneration—A systematic review. Biology.

[11] Hong Y., Li R., Sheng S., Zhou F., Bai L., Su J. (2025). Bone organoid construction and evolution. J. Orthop. Transl..

[12] An J., Yang H., Zhang Q., Liu C., Zhao J., Zhang L., Chen B. (2016). Natural products for treatment of osteoporosis: The effects and mechanisms on promoting osteoblast-mediated bone formation. Life Sci..

[13] Marie P.J., Kassem M. (2011). Osteoblasts in osteoporosis: Past, emerging, and future anabolic targets. Eur. J. Endocrinol..

[14] Zhou X., Cao H., Guo J., Yuan Y., Ni G. (2022). Effects of BMSC-derived EVs on bone metabolism. Pharmaceutics.

[15] Dalle Carbonare L., Innamorati G., Valenti M.T. (2012). Transcription factor Runx2 and its application to bone tissue engineering. Stem Cell Rev. Rep..

[16] Torrecillas-Baena B., Pulido-Escribano V., Dorado G., Gálvez-Moreno M.Á., Camacho-Cardenosa M., Casado-Díaz A. (2023). Clinical potential of mesenchymal stem cell-derived exosomes in bone regeneration. J. Clin. Med..

[17] Hass R., Kasper C., Böhm S., Jacobs R. (2011). Different populations and sources of human mesenchymal stem cells (MSC): A comparison of adult and neonatal tissue-derived MSC. Cell Commun. Signal..

[18] Confalonieri D., Schwab A., Walles H., Ehlicke F. (2018). Advanced therapy medicinal products: A guide for bone marrow-derived MSC application in bone and cartilage tissue engineering. Tissue Eng. Part B Rev..

[19] Li L., Zhou X., Zhang J.-T., Liu A.-F., Zhang C., Han J.-C., Zhang X.-Q., Wu S., Zhang X.-Y., Lv F.-Q. (2021). Exosomal miR-186 derived from BMSCs promote osteogenesis through hippo signaling pathway in postmenopausal osteoporosis. J. Orthop. Surg. Res..

[20] Zuk P.A., Zhu M., Mizuno H., Huang J., Futrell J.W., Katz A.J., Benhaim P., Lorenz H.P., Hedrick M.H. (2001). Multilineage cells from human adipose tissue: Implications for cell-based therapies. Tissue Eng..

[21] Zhang R., Xu Z., Xu S., Li R., Qian H. (2025). Biological Nanotherapeutics Derived From Human Umbilical Cord Mesenchymal Stem Cells: Mechanisms and Translational Potential in Multisystem Therapies for Regeneration and Oncology. Int. J. Nanomed..

[22] Takahashi K., Nakamura M., Okubo C., Kliesmete Z., Ohnuki M., Narita M., Watanabe A., Ueda M., Takashima Y., Hellmann I. (2021). The pluripotent stem cell-specific transcript ESRG is dispensable for human pluripotency. PLoS Genet..

[23] Thanaskody K., Jusop A.S., Tye G.J., Zaman W.S.W.K., Dass S.A., Nordin F. (2022). MSCs vs. iPSCs: Potential in therapeutic applications. Front. Cell Dev. Biol..

[24] Taheri B., Soleimani M., Fekri Aval S., Esmaeili E., Bazi Z., Zarghami N. (2019). Induced pluripotent stem cell-derived extracellular vesicles: A novel approach for cell-free regenerative medicine. J. Cell. Physiol..

[25] Zhu Y., Wang Y., Zhao B., Niu X., Hu B., Li Q., Zhang J., Ding J., Chen Y., Wang Y. (2017). Comparison of exosomes secreted by induced pluripotent stem cell-derived mesenchymal stem cells and synovial membrane-derived mesenchymal stem cells for the treatment of osteoarthritis. Stem Cell Res. Ther..

[26] Imanishi Y., Hata M., Matsukawa R., Aoyagi A., Omi M., Mizutani M., Naruse K., Ozawa S., Honda M., Matsubara T. (2021). Efficacy of extracellular vesicles from dental pulp stem cells for bone regeneration in rat calvarial bone defects. Inflamm. Regen..

[27] Zhou H., Li X., Yin Y., He X.-T., An Y., Tian B.-M., Hong Y.-L., Wu L.-A., Chen F.-M. (2020). The proangiogenic effects of extracellular vesicles secreted by dental pulp stem cells derived from periodontally compromised teeth. Stem Cell Res. Ther..

[28] Jin Q., Li P., Yuan K., Zhao F., Zhu X., Zhang P., Huang Z. (2020). Extracellular vesicles derived from human dental pulp stem cells promote osteogenesis of adipose-derived stem cells via the MAPK pathway. J. Tissue Eng..

[29] Lu Y., Zhao L., Mao J., Liu W., Ma W., Zhao B. (2023). Rab27a-mediated extracellular vesicle secretion contributes to osteogenesis in periodontal ligament-bone niche communication. Sci. Rep..

[30] Lan Q., Cao J., Bi X., Xiao X., Li D., Ai Y. (2023). Curcumin-primed periodontal ligament stem cells-derived extracellular vesicles improve osteogenic ability through the Wnt/β-catenin pathway. Front. Cell Dev. Biol..

[31] Lan Q., Xiao X., Bi X., Gu Y., Ai Y. (2024). Effects of periodontal ligament stem cell-derived exosomes on osteoblastic proliferation, migration, differentiation, apoptosis, and signaling pathways. Oral Dis..

[32] Pranskunas M., Šimoliūnas E., Alksne M., Martin V., Gomes P.S., Puisys A., Kaupinis A., Juodzbalys G. (2021). Assessment of the bone healing process mediated by periosteum-derived mesenchymal stem cells’ secretome and a xenogenic bioceramic—An in vivo study in the rabbit critical size calvarial defect model. Materials.

[33] Tang D., Tang W., Chen H., Liu D., Jiao F. (2024). Synergistic Effects of Icariin and Extracellular Vesicles Derived from Rabbit Synovial Membrane-Derived Mesenchymal Stem Cells on Osteochondral Repair via the Wnt/β-Catenin Pathway. Anal. Cell. Pathol..

[34] Rosu A., Ghaemi B., Bulte J.W., Shakeri-Zadeh A. (2024). Tumor-tropic Trojan horses: Using mesenchymal stem cells as cellular nanotheranostics. Theranostics.

[35] Ding Z., Greenberg Z.F., Serafim M.F., Ali S., Jamieson J.C., Traktuev D.O., March K., He M. (2024). Understanding molecular characteristics of extracellular vesicles derived from different types of mesenchymal stem cells for therapeutic translation. Extracell. Vesicle.

[36] Qiu M., Zhai S., Fu Q., Liu D. (2021). Bone marrow mesenchymal stem cells-derived exosomal microRNA-150-3p promotes osteoblast proliferation and differentiation in osteoporosis. Hum. Gene Ther..

[37] You L., Pan L., Chen L., Gu W., Chen J. (2016). MiR-27a is essential for the shift from osteogenic differentiation to adipogenic differentiation of mesenchymal stem cells in postmenopausal osteoporosis. Cell. Physiol. Biochem..

[38] Wang Y., Zhou X., Wang D. (2022). Mesenchymal stem cell–derived extracellular vesicles inhibit osteoporosis via microRNA-27a-induced inhibition of DKK2-mediated Wnt/β-catenin pathway. Inflammation.

[39] Wei Y., Ma H., Zhou H., Yin H., Yang J., Song Y., Yang B. (2021). miR-424-5p shuttled by bone marrow stem cells-derived exosomes attenuates osteogenesis via regulating WIF1-mediated Wnt/β-catenin axis. Aging.

[40] Al-Sharabi N., Mohamed-Ahmed S., Shanbhag S., Kampleitner C., Elnour R., Yamada S., Rana N., Birkeland E., Tangl S., Gruber R. (2024). Osteogenic human MSC-derived extracellular vesicles regulate MSC activity and osteogenic differentiation and promote bone regeneration in a rat calvarial defect model. Stem Cell Res. Ther..

[41] Tan K.L., Chia W.C., How C.W., Tor Y.S., Show P.L., Looi Q.H.D., Foo J.B. (2021). Benchtop isolation and characterisation of small extracellular vesicles from human mesenchymal stem cells. Mol. Biotechnol..

[42] Batsali A.K., Georgopoulou A., Mavroudi I., Matheakakis A., Pontikoglou C.G., Papadaki H.A. (2020). The role of bone marrow mesenchymal stem cell derived extracellular vesicles (MSC-EVs) in normal and abnormal hematopoiesis and their therapeutic potential. J. Clin. Med..

[43] Liu A., Lin D., Zhao H., Chen L., Cai B., Lin K., Shen S.G. (2021). Optimized BMSC-derived osteoinductive exosomes immobilized in hierarchical scaffold via lyophilization for bone repair through Bmpr2/Acvr2b competitive receptor-activated Smad pathway. Biomaterials.

[44] Wang C., Stöckl S., Li S., Herrmann M., Lukas C., Reinders Y., Sickmann A., Grässel S. (2022). Effects of extracellular vesicles from osteogenic differentiated human BMSCs on osteogenic and adipogenic differentiation capacity of naïve human BMSCs. Cells.

[45] Chen S., Tang Y., Liu Y., Zhang P., Lv L., Zhang X., Jia L., Zhou Y. (2019). Exosomes derived from miR-375-overexpressing human adipose mesenchymal stem cells promote bone regeneration. Cell Prolif..

[46] Ho M.-L., Hsu C.-J., Wu C.-W., Chang L.-H., Chen J.-W., Chen C.-H., Huang K.-C., Chang J.-K., Wu S.-C., Shao P.-L. (2022). Enhancement of osteoblast function through extracellular vesicles derived from adipose-derived stem cells. Biomedicines.

[47] Hu Y., Zhang Y., Ni C.-Y., Chen C.-Y., Rao S.-S., Yin H., Huang J., Tan Y.-J., Wang Z.-X., Cao J. (2020). Human umbilical cord mesenchymal stromal cells-derived extracellular vesicles exert potent bone protective effects by CLEC11A-mediated regulation of bone metabolism. Theranostics.

[48] Gao J., Zhu D., Fan Y., Liu H., Shen Z. (2024). Human umbilical cord mesenchymal stem cells-derived extracellular vesicles for rat jawbone regeneration in periapical periodontitis. ACS Biomater. Sci. Eng..

[49] Chita A. (2025). Engineering the Protein Cargo Levels of Extracellular Vesicles (EVs) Derived from Umbilical Cord Mesenchymal Stem Cells (UC-MSCs).

[50] Liu X., Li Q., Niu X., Hu B., Chen S., Song W., Ding J., Zhang C., Wang Y. (2017). Exosomes secreted from human-induced pluripotent stem cell-derived mesenchymal stem cells prevent osteonecrosis of the femoral head by promoting angiogenesis. Int. J. Biol. Sci..

[51] Gao R., Ye T., Zhu Z., Li Q., Zhang J., Yuan J., Zhao B., Xie Z., Wang Y. (2022). Small extracellular vesicles from iPSC-derived mesenchymal stem cells ameliorate tendinopathy pain by inhibiting mast cell activation. Nanomedicine.

[52] Ye T., Chen Z., Zhang J., Luo L., Gao R., Gong L., Du Y., Xie Z., Zhao B., Li Q. (2023). Large extracellular vesicles secreted by human iPSC-derived MSCs ameliorate tendinopathy via regulating macrophage heterogeneity. Bioact. Mater..

[53] Tertel T., Dittrich R., Arsène P., Jensen A., Giebel B. (2023). EV products obtained from iPSC-derived MSCs show batch-to-batch variations in their ability to modulate allogeneic immune responses in vitro. Front. Cell Dev. Biol..

[54] Chi Y., Liu T., Jin Q., Liu H. (2024). Extracellular vesicles carrying RUNX3 promote differentiation of dental pulp stem cells. Tissue Eng. Regen. Med..

[55] Brunello G., Zanotti F., Trentini M., Zanolla I., Pishavar E., Favero V., Favero R., Favero L., Bressan E., Bonora M. (2022). Exosomes derived from dental pulp stem cells show different angiogenic and osteogenic properties in relation to the age of the donor. Pharmaceutics.

[56] Tang G., Asou Y., Matsumura E., Nakagawa Y., Miyatake K., Katagiri H., Nakamura T., Koga H., Komori K., Sekiya I. (2022). Short cytoplasmic isoform of IL1R1/CD121a mediates IL1β induced proliferation of synovium-derived mesenchymal stem/stromal cells through ERK1/2 pathway. Heliyon.

[57] Jones B.A., Pei M. (2012). Synovium-derived stem cells: A tissue-specific stem cell for cartilage engineering and regeneration. Tissue Eng. Part B Rev..

[58] Sanjurjo-Rodríguez C., Crossland R.E., Reis M., Pandit H., Wang X.-n., Jones E. (2021). Characterization and miRNA profiling of extracellular vesicles from human osteoarthritic subchondral bone multipotential stromal cells (MSCs). Stem Cells Int..

[59] Morito T., Muneta T., Hara K., Ju Y.-J., Mochizuki T., Makino H., Umezawa A., Sekiya I. (2008). Synovial fluid-derived mesenchymal stem cells increase after intra-articular ligament injury in humans. Rheumatology.

[60] Kim S., Koga T., Isobe M., Kern B.E., Yokochi T., Chin Y.E., Karsenty G., Taniguchi T., Takayanagi H. (2003). Stat1 functions as a cytoplasmic attenuator of Runx2 in the transcriptional program of osteoblast differentiation. Genes Dev..

[61] Zhang Y., Cao X., Li P., Fan Y., Zhang L., Ma X., Sun R., Liu Y., Li W. (2021). microRNA-935-modified bone marrow mesenchymal stem cells-derived exosomes enhance osteoblast proliferation and differentiation in osteoporotic rats. Life Sci..

[62] Schunk S.J., Floege J., Fliser D., Speer T. (2021). WNT–β-catenin signalling—A versatile player in kidney injury and repair. Nat. Rev. Nephrol..

[63] Qin Y., Wang L., Gao Z., Chen G., Zhang C. (2016). Bone marrow stromal/stem cell-derived extracellular vesicles regulate osteoblast activity and differentiation in vitro and promote bone regeneration in vivo. Sci. Rep..

[64] Liu T., Hu W., Zou X., Xu J., He S., Chang L., Li X., Yin Y., Tian M., Li Z. (2020). Human periodontal ligament stem cell-derived exosomes promote bone regeneration by altering MicroRNA profiles. Stem Cells Int..

[65] Lu H., Mu Q., Ku W., Zheng Y., Yi P., Lin L., Li P., Wang B., Wu J., Yu D. (2024). Functional extracellular vesicles from SHEDs combined with gelatin methacryloyl promote the odontogenic differentiation of DPSCs for pulp regeneration. J. Nanobiotechnol..

[66] Wang M., Li J., Ye Y., He S., Song J. (2020). SHED-derived conditioned exosomes enhance the osteogenic differentiation of PDLSCs via Wnt and BMP signaling in vitro. Differentiation.

[67] Hu X., Zhong Y., Kong Y., Chen Y., Feng J., Zheng J. (2019). Lineage-specific exosomes promote the odontogenic differentiation of human dental pulp stem cells (DPSCs) through TGFβ1/smads signaling pathway via transfer of microRNAs. Stem Cell Res. Ther..

[68] Jiang L., Tian L., Zhang C. (2018). Bone marrow stem cells-derived exosomes extracted from osteoporosis patients inhibit osteogenesis via microRNA-21/SMAD7. Eur. Rev. Med. Pharmacol. Sci..

[69] Yang B.-c., Kuang M.-j., Kang J.-y., Zhao J., Ma J.-x., Ma X.-l. (2020). Human umbilical cord mesenchymal stem cell-derived exosomes act via the miR-1263/Mob1/Hippo signaling pathway to prevent apoptosis in disuse osteoporosis. Biochem. Biophys. Res. Commun..

[70] Gao Y., Li X., Ding Y., Wang Y., Du J., Chen Y., Xu J., Liu Y. (2025). MiR-451a-Enriched Small Extracellular Vesicles Derived from Mg2+-Activated DPSCs Induce Vascularized Bone Regeneration through the AKT/eNOS/NO Axis. ACS Appl. Mater. Interfaces.

[71] Li R., Li D., Wang H., Chen K., Wang S., Xu J., Ji P. (2022). Exosomes from adipose-derived stem cells regulate M1/M2 macrophage phenotypic polarization to promote bone healing via miR-451a/MIF. Stem Cell Res. Ther..

[72] Li Z., Yu Q., Cui X., Wang Y., Xu R., Lu R., Chen J., Zhou X., Zhang C., Li L. (2025). Exosomes from young plasma stimulate the osteogenic differentiation and prevent osteoporosis via miR-142-5p. Bioact. Mater..

[73] Wang L., Wei X., He X., Xiao S., Shi Q., Chen P., Lee J., Guo X., Liu H., Fan Y. (2024). Osteoinductive dental pulp stem cell-derived extracellular vesicle-loaded multifunctional hydrogel for bone regeneration. ACS Nano.

[74] Xie L., Ren X., Yang Z., Zhou T., Zhang M., An W., Guan Z. (2024). Exosomal circ_0000722 derived from periodontal ligament stem cells undergoing osteogenic differentiation promotes osteoclastogenesis. Int. Immunopharmacol..

[75] Zhang S., Wang S., Chen J., Cui Y., Lu X., Xiong S., Yue C., Yang B. (2024). Human dental pulp stem cell-derived exosomes decorated titanium scaffolds for promoting bone regeneration. Colloids Surf. B Biointerfaces.

[76] Liu X., Li Z., Fu J., Wang R., He J., Yao J., Ye Q., He Y. (2025). Tailored Extracellular Vesicles from Dental Stem Cells: Advances in Specific Modifications for Enhanced Therapeutic Applications. Int. J. Nanomed..

[77] Cui H., Wang Y., Wang D., Zhang H., Zhou L., Qin M., Li G., Ma T., Li Y., Dong B. (2025). Mechanical stimulation of extracellular vesicles secreted by bone marrow mesenchymal stem cells promotes osteoblast proliferation and differentiation by activating the Wnt/β-catenin signaling pathway. Connect. Tissue Res..

[78] Li L., Cheng L., Du Y., Zhang Y., Wang Z., Nie Y., Long J., Li C., Zhang Y., Lai Y. (2025). Exosomes Derived from Mg-Preconditioned Bone Mesenchymal Stem Cells Promote Angiogenesis and Osteogenesis for Osteonecrosis Treatment. Materials.

[79] Rajan Unnithan A., Man K., Kritika, Gethings L.A., Hughes C.J., Keenan A., Heaney L., Cox S.C., Davies O.G., El Haj A.J. (2025). Engineering Extracellular Vesicle Production through Magnetic Ion Channel Activation for Bone Regeneration. bioRxiv.

[80] Liu D., Shi B., Zhou W., Tao G. (2023). Exosomes from hypoxia-conditioned apical papilla stem cells accelerate angiogenesis in vitro through Notch/JAG1/VEGF signaling. Tissue Cell.

[81] Gao Y., Yuan Z., Yuan X., Wan Z., Yu Y., Zhan Q., Zhao Y., Han J., Huang J., Xiong C. (2022). Bioinspired porous microspheres for sustained hypoxic exosomes release and vascularized bone regeneration. Bioact. Mater..

[82] Chen Y., Liu M., Niu Y., Wang Y. (2020). Romance of the three kingdoms in hypoxia: HIFs, epigenetic regulators, and chromatin reprogramming. Cancer Lett..

[83] Gonzalez-King H., García N.A., Ontoria-Oviedo I., Ciria M., Montero J.A., Sepúlveda P. (2017). Hypoxia inducible factor-1α potentiates jagged 1-mediated angiogenesis by mesenchymal stem cell-derived exosomes. Stem Cells.

[84] Gómez-Ferrer M., Villanueva-Badenas E., Sánchez-Sánchez R., Sánchez-López C.M., Baquero M.C., Sepúlveda P., Dorronsoro A. (2021). HIF-1α and pro-inflammatory signaling improves the immunomodulatory activity of MSC-derived extracellular vesicles. Int. J. Mol. Sci..

[85] Wang H., Zhao H., Chen Z., Cai X., Wang X., Zhou P., Tang Y., Ying T., Zhang X., Shen Y. (2024). Hypoxic bone mesenchymal stem cell-derived exosomes direct schwann cells proliferation, migration, and paracrine to accelerate facial nerve regeneration via circRNA_Nkd2/miR-214-3p/MED19 Axis. Int. J. Nanomed..

[86] Biswas S., Gangadaran P., Dhara C., Ghosh S., Phadikar S.D., Chakraborty A., Mahajan A.A., Mondal R., Chattopadhyay D., Banerjee T. (2025). Extracellular Vesicles in Osteogenesis: A Comprehensive Review of Mechanisms and Therapeutic Potential for Bone Regeneration. Curr. Issues Mol. Biol..

[87] Jiang S., Wang M., He J. (2021). A review of biomimetic scaffolds for bone regeneration: Toward a cell-free strategy. Bioeng. Transl. Med..

[88] Sun X., Mao Y., Liu B., Gu K., Liu H., Du W., Li R., Zhang J. (2023). Mesenchymal stem cell-derived exosomes enhance 3D-printed scaffold functions and promote alveolar bone defect repair by enhancing angiogenesis. J. Pers. Med..

[89] Diomede F., D’aurora M., Gugliandolo A., Merciaro I., Ettorre V., Bramanti A., Piattelli A., Gatta V., Mazzon E., Fontana A. (2018). A novel role in skeletal segment regeneration of extracellular vesicles released from periodontal-ligament stem cells. Int. J. Nanomed..

[90] Diomede F., Gugliandolo A., Cardelli P., Merciaro I., Ettorre V., Traini T., Bedini R., Scionti D., Bramanti A., Nanci A. (2018). Three-dimensional printed PLA scaffold and human gingival stem cell-derived extracellular vesicles: A new tool for bone defect repair. Stem Cell Res. Ther..

[91] Pizzicannella J., Gugliandolo A., Orsini T., Fontana A., Ventrella A., Mazzon E., Bramanti P., Diomede F., Trubiani O. (2019). Engineered extracellular vesicles from human periodontal-ligament stem cells increase VEGF/VEGFR2 expression during bone regeneration. Front. Physiol..

[92] Kang Y., Xu C., Meng L.a., Dong X., Qi M., Jiang D. (2022). Exosome-functionalized magnesium-organic framework-based scaffolds with osteogenic, angiogenic and anti-inflammatory properties for accelerated bone regeneration. Bioact. Mater..

[93] Li G., Zhang Y., Wu J., Yang R., Sun Q., Xu Y., Wang B., Cai M., Xu Y., Zhuang C. (2023). Adipose stem cells-derived exosomes modified gelatin sponge promotes bone regeneration. Front. Bioeng. Biotechnol..

[94] Li Q., Yu H., Zhao F., Cao C., Wu T., Fan Y., Ao Y., Hu X. (2023). 3D printing of microenvironment-specific bioinspired and exosome-reinforced hydrogel scaffolds for efficient cartilage and subchondral bone regeneration. Adv. Sci..

[95] Vonk L.A., van Dooremalen S.F., Liv N., Klumperman J., Coffer P.J., Saris D.B., Lorenowicz M.J. (2018). Mesenchymal stromal/stem cell-derived extracellular vesicles promote human cartilage regeneration in vitro. Theranostics.

[96] de Windt T.S., Saris D.B., Slaper-Cortenbach I.C., van Rijen M.H., Gawlitta D., Creemers L.B., de Weger R.A., Dhert W.J., Vonk L.A. (2015). Direct cell–cell contact with chondrocytes is a key mechanism in multipotent mesenchymal stromal cell-mediated chondrogenesis. Tissue Eng. Part A.

[97] Wang L., Wang J., Zhou X., Sun J., Zhu B., Duan C., Chen P., Guo X., Zhang T., Guo H. (2020). A new self-healing hydrogel containing hucMSC-derived exosomes promotes bone regeneration. Front. Bioeng. Biotechnol..

[98] Eichholz K.F., Woods I., Riffault M., Johnson G.P., Corrigan M., Lowry M.C., Shen N., Labour M.-N., Wynne K., O’Driscoll L. (2020). Human bone marrow stem/stromal cell osteogenesis is regulated via mechanically activated osteocyte-derived extracellular vesicles. Stem Cells Transl. Med..

[99] Du Z., Rizzo S.A., Sarrafian T.L., Bagwell M.S., Mahlberg R.C., Amontree A., Schiebel P., Tauferner D.M., LeBrasseur Z.S., Witt T.A. (2025). Engineered BMP2/BMP7 extracellular vesicles induce autocrine BMP release driving SMAD phosphorylation to promote bone formation. NPJ Regen. Med..

[100] Zhao B., Chen Q., Zhao L., Mao J., Huang W., Han X., Liu Y. (2022). Periodontal ligament stem cell-derived small extracellular vesicles embedded in matrigel enhance bone repair through the adenosine receptor signaling pathway. Int. J. Nanomed..

[101] Qi X., Zhang J., Yuan H., Xu Z., Li Q., Niu X., Hu B., Wang Y., Li X. (2016). Exosomes secreted by human-induced pluripotent stem cell-derived mesenchymal stem cells repair critical-sized bone defects through enhanced angiogenesis and osteogenesis in osteoporotic rats. Int. J. Biol. Sci..

[102] Xie H., Wang Z., Zhang L., Lei Q., Zhao A., Wang H., Li Q., Chen Z., Zhang W. (2016). Development of an angiogenesis-promoting microvesicle-alginate-polycaprolactone composite graft for bone tissue engineering applications. PeerJ.

[103] Almeria C., Weiss R., Keck M., Weber V., Kasper C., Egger D. (2024). Dynamic cultivation of human mesenchymal stem/stromal cells for the production of extracellular vesicles in a 3D bioreactor system. Biotechnol. Lett..

[104] Gobin J., Muradia G., Mehic J., Westwood C., Couvrette L., Stalker A., Bigelow S., Luebbert C.C., Bissonnette F.S.-D., Johnston M.J. (2021). Hollow-fiber bioreactor production of extracellular vesicles from human bone marrow mesenchymal stromal cells yields nanovesicles that mirrors the immuno-modulatory antigenic signature of the producer cell. Stem Cell Res. Ther..

[105] Jakl V., Ehmele M., Winkelmann M., Ehrenberg S., Eiseler T., Friemert B., Rojewski M.T., Schrezenmeier H. (2023). A novel approach for large-scale manufacturing of small extracellular vesicles from bone marrow-derived mesenchymal stromal cells using a hollow fiber bioreactor. Front. Bioeng. Biotechnol..

[106] Bahmaee H., Owen R., Boyle L., Perrault C.M., Garcia-Granada A.A., Reilly G.C., Claeyssens F. (2020). Design and evaluation of an osteogenesis-on-a-chip microfluidic device incorporating 3D cell culture. Front. Bioeng. Biotechnol..

[107] Yang J., Duan P., Liu Q., Yu H., Fang F., Liu X. (2024). Microfluidic bone chip to study osteogenesis of porous substrate topographies in normal and osteoporotic microenvironments. Eur. Cells Mater..

[108] Vis M., Zhao F., Bodelier E., Bood C., Bulsink J., Van Doeselaar M., Amirabadi H.E., Ito K., Hofmann S. (2023). Osteogenesis and osteoclastogenesis on a chip: Engineering a self-assembling 3D coculture. Bone.

[109] Maritan S.M., Lian E.Y., Mulligan L.M. (2017). An efficient and flexible cell aggregation method for 3D spheroid production. J. Vis. Exp. JoVE.

[110] Olivares A.L., Marsal È., Planell J.A., Lacroix D. (2009). Finite element study of scaffold architecture design and culture conditions for tissue engineering. Biomaterials.

[111] Zhao D., Saiding Q., Li Y., Tang Y., Cui W. (2024). Bone organoids: Recent advances and future challenges. Adv. Healthc. Mater..

[112] Bai L., Zhou D., Li G., Liu J., Chen X., Su J. (2024). Engineering bone/cartilage organoids: Strategy, progress, and application. Bone Res..

[113] Liu H., Su J. (2023). Organoid extracellular vesicle-based therapeutic strategies for bone therapy. Biomater. Transl..

[114] Wang L., Wang D., Ye Z., Xu J. (2023). Engineering extracellular vesicles as delivery systems in therapeutic applications. Adv. Sci..

[115] Abdollahi S. (2021). Extracellular vesicles from organoids and 3D culture systems. Biotechnol. Bioeng..

[116] Zheng J., Yao Z., Xue L., Wang D., Tan Z. (2022). The role of immune cells in modulating chronic inflammation and osteonecrosis. Front. Immunol..

[117] Vig S., Fernandes M.H. (2022). Bone cell exosomes and emerging strategies in bone engineering. Biomedicines.

[118] Chen C.-Y., Du W., Rao S.-S., Tan Y.-J., Hu X.-K., Luo M.-J., Ou Q.-F., Wu P.-F., Qing L.-M., Cao Z.-M. (2020). Extracellular vesicles from human urine-derived stem cells inhibit glucocorticoid-induced osteonecrosis of the femoral head by transporting and releasing pro-angiogenic DMBT1 and anti-apoptotic TIMP1. Acta Biomater..

[119] Huang S., Li Y., Wu P., Xiao Y., Duan N., Quan J., Du W. (2020). microRNA-148a-3p in extracellular vesicles derived from bone marrow mesenchymal stem cells suppresses SMURF1 to prevent osteonecrosis of femoral head. J. Cell. Mol. Med..

[120] Watanabe J., Sakai K., Urata Y., Toyama N., Nakamichi E., Hibi H. (2020). Extracellular vesicles of stem cells to prevent BRONJ. J. Dent. Res..

[121] Huang J., Wang L., Tian W. (2021). Small extracellular vesicles derived from adipose tissue prevent bisphosphonate-related osteonecrosis of the jaw by promoting angiogenesis. Int. J. Nanomed..

[122] Zhang G., Liu R., Dang X., Liu J., Jiao H. (2021). Experimental study on improvement of osteonecrosis of femoral head with exosomes derived from miR-27a-overexpressing vascular endothelial cells. Chin. J. Reparative Reconstr. Surg..

[123] Li H., Liu D., Li C., Zhou S., Tian D., Xiao D., Zhang H., Gao F., Huang J. (2017). Exosomes secreted from mutant-HIF-1α-modified bone-marrow-derived mesenchymal stem cells attenuate early steroid-induced avascular necrosis of femoral head in rabbit. Cell Biol. Int..

[124] Nan K., Zhang Y., Zhang X., Li D., Zhao Y., Jing Z., Liu K., Shang D., Geng Z., Fan L. (2021). Exosomes from miRNA-378-modified adipose-derived stem cells prevent glucocorticoid-induced osteonecrosis of the femoral head by enhancing angiogenesis and osteogenesis via targeting miR-378 negatively regulated suppressor of fused (Sufu). Stem Cell Res. Ther..

[125] Li Y., Ma X., Dong B., Li Y., Liang Z. (2024). Network meta-analysis of invasive treatment for early-stage osteonecrosis of the femoral head. J. Orthop. Surg. Res..

[126] Yuan F.-L., Wu Q.-Y., Miao Z.-N., Xu M.-H., Xu R.-S., Jiang D.-L., Ye J.-X., Chen F.-H., Zhao M.-D., Wang H.-J. (2018). Osteoclast-derived extracellular vesicles: Novel regulators of osteoclastogenesis and osteoclast–osteoblasts communication in bone remodeling. Front. Physiol..

[127] Shi H., Jiang X., Xu C., Cheng Q. (2022). MicroRNAs in serum exosomes as circulating biomarkers for postmenopausal osteoporosis. Front. Endocrinol..

[128] Li F., Zhao X., Zhang Y., Zhuang Q., Wang S., Fang X., Xu T., Li X., Chen G. (2024). Exosomal circFAM63Bsuppresses bone regeneration of postmenopausal osteoporosis via regulating miR-578/HMGA2 axis. J. Orthop. Res..

[129] Luo Z.-W., Liu Y.-W., Rao S.-S., Yin H., Huang J., Chen C.-Y., Hu Y., Zhang Y., Tan Y.-J., Yuan L.-Q. (2019). Aptamer-functionalized exosomes from bone marrow stromal cells target bone to promote bone regeneration. Nanoscale.

[130] Huang B., Su Y., Shen E., Song M., Liu D., Qi H. (2021). Extracellular vesicles from GPNMB-modified bone marrow mesenchymal stem cells attenuate bone loss in an ovariectomized rat model. Life Sci..

[131] Wang X., Zou C., Hou C., Bian Z., Jiang W., Li M., Zhu L. (2023). Extracellular vesicles from bone marrow mesenchymal stem cells alleviate osteoporosis in mice through USP7-mediated YAP1 protein stability and the Wnt/β-catenin pathway. Biochem. Pharmacol..

[132] Deluca A., Wagner A., Heimel P., Deininger C., Wichlas F., Redl H., Rohde E., Tempfer H., Gimona M., Traweger A. (2024). Synergistic effect of umbilical cord extracellular vesicles and rhBMP-2 to enhance the regeneration of a metaphyseal femoral defect in osteoporotic rats. Stem Cell Res. Ther..

[133] Wang Y., Yao J., Cai L., Liu T., Wang X., Zhang Y., Zhou Z., Li T., Liu M., Lai R. (2020). Bone-targeted extracellular vesicles from mesenchymal stem cells for osteoporosis therapy. Int. J. Nanomed..

[134] Cheng Y., Zhu Y., Liu Y., Liu X., Ding Y., Li D., Zhang X., Liu Y. (2024). Tailored apoptotic vesicles promote bone regeneration by releasing the osteoinductive brake. Int. J. Oral Sci..

[135] Lener T., Gimona M., Aigner L., Börger V., Buzas E., Camussi G., Chaput N., Chatterjee D., Court F.A., Portillo H.A.d. (2015). Applying extracellular vesicles based therapeutics in clinical trials–an ISEV position paper. J. Extracell. Vesicles.

[136] Wang T., Liu K., Wang J., Xiang G., Hu X., Bai H., Lei W., Tao T.H., Feng Y. (2023). Spatiotemporal regulation of injectable heterogeneous silk gel scaffolds for accelerating guided vertebral repair. Adv. Healthc. Mater..

[137] Qayoom I., Teotia A.K., Kumar A. (2019). Nanohydroxyapatite based ceramic carrier promotes bone formation in a femoral neck canal defect in osteoporotic rats. Biomacromolecules.

[138] Negri S., Wang Y., Sono T., Lee S., Hsu G.C.-Y., Xu J., Meyers C.A., Qin Q., Broderick K., Witwer K.W. (2020). Human perivascular stem cells prevent bone graft resorption in osteoporotic contexts by inhibiting osteoclast formation. Stem Cells Transl. Med..

[139] Teotia A.K., Qayoom I., Singh P., Mishra A., Jaiman D., Seppälä J., Lidgren L., Kumar A. (2021). Exosome-functionalized ceramic bone substitute promotes critical-sized bone defect repair in rats. ACS Appl. Bio Mater..

[140] Mizukami Y., Kawao N., Takafuji Y., Ohira T., Okada K., Jo J.-I., Tabata Y., Kaji H. (2023). Matrix vesicles promote bone repair after a femoral bone defect in mice. PLoS ONE.

[141] Man K., Brunet M.Y., Federici A.S., Hoey D.A., Cox S.C. (2022). An ECM-mimetic hydrogel to promote the therapeutic efficacy of osteoblast-derived extracellular vesicles for bone regeneration. Front. Bioeng. Biotechnol..

[142] Chen M., Li Y., Zhang M., Ge S., Feng T., Chen R., Shen J., Li R., Wang Z., Xie Y. (2024). Histone deacetylase inhibition enhances extracellular vesicles from muscle to promote osteogenesis via miR-873-3p. Signal Transduct. Target. Ther..

[143] Liu K., Sakai K., Watanabe J., Dong J., Maruyama H., Li X., Hibi H. (2024). Conditioned medium of human mesenchymal stem cells affects stem cell senescence in osteoporosis. Biochem. Biophys. Res. Commun..

[144] Li X., Chen R., Li Y., Wang P., Cui Y., Yang L., Zhu X., Zhang R. (2021). miR-27a-5p—Abundant small extracellular vesicles derived from Epimedium-preconditioned bone mesenchymal stem cells stimulate osteogenesis by targeting Atg4B-mediated autophagy. Front. Cell Dev. Biol..

[145] Na W., Kang M.-K., Park S.-H., Kim D.Y., Oh S.Y., Oh M.-S., Park S., Kang I.-J., Kang Y.-H. (2021). Aesculetin accelerates osteoblast differentiation and matrix-vesicle-mediated mineralization. Int. J. Mol. Sci..

[146] Zhan W., Deng M., Huang X., Xie D., Gao X., Chen J., Shi Z., Lu J., Lin H., Li P. (2023). Pueraria lobata-derived exosome-like nanovesicles alleviate osteoporosis by enhacning autophagy. J. Control. Release.

[147] Hwang J.-H., Park Y.-S., Kim H.-S., Kim D.-h., Lee S.-H., Lee C.-H., Lee S.-H., Kim J.-E., Lee S., Kim H.M. (2023). Yam-derived exosome-like nanovesicles stimulate osteoblast formation and prevent osteoporosis in mice. J. Control. Release.

[148] Go G., Jeon J., Lee G., Lee J.H., Lee S.H. (2021). Bovine milk extracellular vesicles induce the proliferation and differentiation of osteoblasts and promote osteogenesis in rats. J. Food Biochem..

[149] Dong M., Shi C., Yu X., Yang Q., Wu S., Liu R., Liu T., Wang L., Niu W. (2022). Milk-derived small extracellular vesicles: Nanomaterials to promote bone formation. J. Nanobiotechnol..

[150] Zheng G., Ma H.-W., Xiang G.-H., He G.-L., Cai H.-C., Dai Z.-H., Chen Y.-L., Lin Y., Xu H.-Z., Ni W.-F. (2022). Bone-targeting delivery of platelet lysate exosomes ameliorates glucocorticoid-induced osteoporosis by enhancing bone-vessel coupling. J. Nanobiotechnol..

[151] Xie X., Cheng P., Hu L., Zhou W., Zhang D., Knoedler S., Liu G., Xiong Y., Xue H., Hu Y. (2024). Bone-targeting engineered small extracellular vesicles carrying anti-miR-6359-CGGGAGC prevent valproic acid-induced bone loss. Signal Transduct. Target. Ther..

[152] Roddy E., DeBaun M.R., Daoud-Gray A., Yang Y.P., Gardner M.J. (2018). Treatment of critical-sized bone defects: Clinical and tissue engineering perspectives. Eur. J. Orthop. Surg. Traumatol..

[153] Jia Y., Qiu S., Xu J., Kang Q., Chai Y. (2020). Exosomes secreted by young mesenchymal stem cells promote new bone formation during distraction osteogenesis in older rats. Calcif. Tissue Int..

[154] Yu L., Dou G., Kuang H., Bao L., Liu H., Ye Q., Wang Z., Yang X., Ren L., Li Z. (2024). Apoptotic extracellular vesicles induced endothelial cell-mediated autologous stem cell recruitment dominates allogeneic stem cell therapeutic mechanism for bone repair. ACS Nano.

[155] Sun R., Xu S., Wang Z. (2019). Rat sinus mucosa-and periosteum-derived exosomes accelerate osteogenesis. J. Cell. Physiol..

[156] Cao Z., Wu Y., Yu L., Zou L., Yang L., Lin S., Wang J., Yuan Z., Dai J. (2021). Exosomal miR-335 derived from mature dendritic cells enhanced mesenchymal stem cell-mediated bone regeneration of bone defects in athymic rats. Mol. Med..

[157] Kang M., Huang C.-C., Lu Y., Shirazi S., Gajendrareddy P., Ravindran S., Cooper L.F. (2020). Bone regeneration is mediated by macrophage extracellular vesicles. Bone.

[158] Xu J., Wang Y., Hsu C.-Y., Gao Y., Meyers C.A., Chang L., Zhang L., Broderick K., Ding C., Peault B. (2019). Human perivascular stem cell-derived extracellular vesicles mediate bone repair. Elife.

[159] Zheng J., He J., Wu J., Yu Y., Fu Y., Yin S., Li K., Li Y., Cai L., Du Y. (2025). Polyphenol-Mediated Electroactive Hydrogel with Armored Exosomes Delivery for Bone Regeneration. ACS Nano.

[160] Gao Y., Yuan X., Gu R., Wang N., Ren H., Song R., Wan Z., Huang J., Yi K., Xiong C. (2025). Affinity Modifications of Porous Microscaffolds Impact Bone Regeneration by Modulating the Delivery Kinetics of Small Extracellular Vesicles. ACS Nano.

[161] Pan S., Yin Z., Shi C., Xiu H., Wu G., Heng Y., Zhu Z., Zhang J., Gui J., Yu Z. (2024). Multifunctional Injectable Hydrogel Microparticles Loaded With miR-29a Abundant BMSCs Derived Exosomes Enhanced Bone Regeneration by Regulating Osteogenesis and Angiogenesis. Small.

[162] Shou J., Li S., Shi W., Zhang S., Zeng Z., Guo Z., Ye Z., Wen Z., Qiu H., Wang J. (2023). 3WJ RNA nanoparticles-aptamer functionalized exosomes from M2 macrophages target BMSCs to promote the healing of bone fractures. Stem Cells Transl. Med..

[163] Zhang Y., Hao Z., Wang P., Xia Y., Wu J., Xia D., Fang S., Xu S. (2019). Exosomes from human umbilical cord mesenchymal stem cells enhance fracture healing through HIF-1α-mediated promotion of angiogenesis in a rat model of stabilized fracture. Cell Prolif..

[164] Lu J., Wang Q.-Y., Sheng J.-G. (2019). Exosomes in the repair of bone defects: Next-generation therapeutic tools for the treatment of nonunion. BioMed Res. Int..

[165] Hao Z.C., Lu J., Wang S.Z., Wu H., Zhang Y.T., Xu S.G. (2017). Stem cell-derived exosomes: A promising strategy for fracture healing. Cell Prolif..

[166] Liu Y., Lin S., Xu Z., Wu Y., Wang G., Yang G., Cao L., Chang H., Zhou M., Jiang X. (2024). High-Performance Hydrogel-Encapsulated Engineered Exosomes for Supporting Endoplasmic Reticulum Homeostasis and Boosting Diabetic Bone Regeneration. Adv. Sci..

[167] Tao S.-C., Li X.-R., Wei W.-J., Wei Z.-Y., Zhang C.-R., Wang F., Dawes H., Guo S.-C. (2022). Polymeric coating on β-TCP scaffolds provides immobilization of small extracellular vesicles with surface-functionalization and ZEB1-Loading for bone defect repair in diabetes mellitus. Biomaterials.

[168] Jing X., Wang S., Tang H., Li D., Zhou F., Xin L., He Q., Hu S., Zhang T., Chen T. (2022). Dynamically bioresponsive DNA hydrogel incorporated with dual-functional stem cells from apical papilla-derived exosomes promotes diabetic bone regeneration. ACS Appl. Mater. Interfaces.

[169] Yang T., Dong Y., Wan J., Liu X., Liu Y., Huang J., Zhou J., Xiao H., Tang L., Wang Y. (2023). Sustained release of BMSC-EVs from 3D printing gel/HA/nHAP scaffolds for promoting bone regeneration in diabetic rats. Adv. Healthc. Mater..

[170] Zhang D., Wu Y., Li Z., Chen H., Huang S., Jian C., Yu A. (2021). MiR-144-5p, an exosomal miRNA from bone marrow-derived macrophage in type 2 diabetes, impairs bone fracture healing via targeting Smad1. J. Nanobiotechnol..

[171] Lu W., Yan J., Wang C., Qin W., Han X., Qin Z., Wei Y., Xu H., Gao J., Gao C. (2024). Interorgan communication in neurogenic heterotopic ossification: The role of brain-derived extracellular vesicles. Bone Res..

[172] Song W., Ma Z., Wang X., Wang Y., Wu D., Wang C., He D., Kong L., Yu W., Li J.J. (2023). Macroporous granular hydrogels functionalized with aligned architecture and small extracellular vesicles stimulate osteoporotic tendon-to-bone healing. Adv. Sci..

[173] Ren Y., Zhang S., Wang Y., Jacobson D.S., Reisdorf R.L., Kuroiwa T., Behfar A., Moran S.L., Steinmann S.P., Zhao C. (2021). Effects of purified exosome product on rotator cuff tendon-bone healing in vitro and in vivo. Biomaterials.

[174] Cai J., Xu J., Ye Z., Wang L., Zheng T., Zhang T., Li Y., Jiang J., Zhao J. (2023). Exosomes derived from kartogenin-preconditioned mesenchymal stem cells promote cartilage formation and collagen maturation for enthesis regeneration in a rat model of chronic rotator cuff tear. Am. J. Sports Med..

[175] Jenner F., Wagner A., Gerner I., Ludewig E., Trujanovic R., Rohde E., von Rechenberg B., Gimona M., Traweger A. (2023). Evaluation of the potential of umbilical cord mesenchymal stromal cell–derived small extracellular vesicles to improve rotator cuff healing: A pilot ovine study. Am. J. Sports Med..

[176] Xue Y., Riva N., Zhao L., Shieh J.-s., Chin Y.-T., Gatt A., Guo J.J. (2023). Recent advances of exosomes in soft tissue injuries in sports medicine: A critical review on biological and biomaterial applications. J. Control. Release.

[177] Cosenza S., Ruiz M., Toupet K., Jorgensen C., Noël D. (2017). Mesenchymal stem cells derived exosomes and microparticles protect cartilage and bone from degradation in osteoarthritis. Sci. Rep..

[178] Sankaranarayanan J., Kim H.K., Kang J.Y., Kuppa S.S., Yang H.Y., Seon J.K. (2025). Comparative Efficacy of Exosomes Derived from Different Mesenchymal Stem Cell Sources in Osteoarthritis Models: An In Vitro and Ex Vivo Analysis. Int. J. Mol. Sci..

[179] Xu C., Mi Z., Dong Z., Chen X., Ji G., Kang H., Li K., Zhao B., Wang F. (2023). Platelet-derived exosomes alleviate knee osteoarthritis by attenuating cartilage degeneration and subchondral bone loss. Am. J. Sports Med..

[180] Zhang Y., Qi G., Yan Y., Wang C., Wang Z., Jiang C., Jiang Z., Ma T., Zhang C., Yan Z. (2023). Exosomes derived from bone marrow mesenchymal stem cells pretreated with decellularized extracellular matrix enhance the alleviation of osteoarthritis through miR-3473b/phosphatase and tensin homolog axis. J. Gene Med..

[181] Trivanovic D., Volkmann N., Stoeckl M., Tertel T., Rudert M., Giebel B., Herrmann M. (2023). Enhancement of immunosuppressive activity of mesenchymal stromal cells by platelet-derived factors is accompanied by apoptotic priming. Stem Cell Rev. Rep..

[182] Chen Z., Ding W., Duan P., Lv X., Feng Y., Yin Z., Luo Z., Li Z., Zhang H., Zhou T. (2023). HWJMSC-derived extracellular vesicles ameliorate IL-1β-induced chondrocyte injury through regulation of the BMP2/RUNX2 axis via up-regulation TFRC. Cell. Signal..

[183] Zhang S., Chu W., Lai R., Lim S., Hui J., Toh W. (2016). Exosomes derived from human embryonic mesenchymal stem cells promote osteochondral regeneration. Osteoarthr. Cartil..

[184] Niedermair T., Lukas C., Li S., Stöckl S., Craiovan B., Brochhausen C., Federlin M., Herrmann M., Grässel S. (2020). Influence of extracellular vesicles isolated from osteoblasts of patients with cox-arthrosis and/or osteoporosis on metabolism and osteogenic differentiation of BMSCs. Front. Bioeng. Biotechnol..

[185] Liu Y., Zeng Y., Si H.-B., Tang L., Xie H.-Q., Shen B. (2022). Exosomes derived from human urine–derived stem cells overexpressing miR-140-5p alleviate knee osteoarthritis through downregulation of VEGFA in a rat model. Am. J. Sports Med..

[186] Li H., Fan X.-L., Wang Y.-N., Lu W., Wang H., Liao R., Zeng M., Yang J.-X., Hu Y., Xie J. (2021). Extracellular vesicles from human urine-derived stem cells ameliorate particulate polyethylene-induced osteolysis. Int. J. Nanomed..

[187] Xu H., Chai Q., Xu X., Li Z., Bao W., Man Z., Li W. (2022). Exosome-functionalized Ti6Al4V scaffolds promoting osseointegration by modulating endogenous osteogenesis and osteoimmunity. ACS Appl. Mater. Interfaces.

[188] Jin S., Wen J., Zhang Y., Mou P., Luo Z., Cai Y., Chen A., Fu X., Meng W., Zhou Z. (2024). M2 macrophage-derived exosome-functionalized topological scaffolds regulate the foreign body response and the coupling of angio/osteoclasto/osteogenesis. Acta Biomater..

[189] Olaechea A., Benabdellah K., Vergara-Buenaventura A., Gómez-Melero S., Cafferata E.A., Meza-Mauricio J., Padial-Molina M., Galindo-Moreno P. (2023). Preclinical evidence for the use of oral mesenchymal stem cell-derived extracellular vesicles in bone regenerative therapy: A systematic review. Stem Cells Transl. Med..

[190] Guo S., Gu J., Ma J., Xu R., Wu Q., Meng L., Liu H., Li L., Xu Y. (2021). GATA4-driven miR-206-3p signatures control orofacial bone development by regulating osteogenic and osteoclastic activity. Theranostics.

[191] Yi G., Zhang S., Ma Y., Yang X., Huo F., Chen Y., Yang B., Tian W. (2022). Matrix vesicles from dental follicle cells improve alveolar bone regeneration via activation of the PLC/PKC/MAPK pathway. Stem Cell Res. Ther..

[192] Jiang S., Xu L. (2020). Exosomes from gingival mesenchymal stem cells enhance migration and osteogenic differentiation of pre-osteoblasts. Die Pharm.-Int. J. Pharm. Sci..

[193] Wang W., Qiao S.-C., Wu X.-B., Sun B., Yang J.-G., Li X., Zhang X., Qian S.-J., Gu Y.-X., Lai H.-C. (2021). Circ_0008542 in osteoblast exosomes promotes osteoclast-induced bone resorption through m6A methylation. Cell Death Dis..

[194] Maiborodin I., Shevela A., Matveeva V., Morozov V., Toder M., Krasil’nikov S., Koryakina A., Shevela A., Yanushevich O. (2021). First experimental study of the influence of Extracellular vesicles derived from multipotent stromal cells on Osseointegration of Dental implants. Int. J. Mol. Sci..

[195] Yang Y., Wang J., Lin X., Zhang Z., Zhang M., Tang C., Kou X., Deng F. (2024). TNF-α-licensed exosome-integrated titaniumaccelerated T2D osseointegration by promoting autophagy-regulated M2 macrophage polarization. Biochem. Biophys. Res. Commun..

[196] Liu L., Guo S., Shi W., Liu Q., Huo F., Wu Y., Tian W. (2021). Bone marrow mesenchymal stem cell-derived small extracellular vesicles promote periodontal regeneration. Tissue Eng. Part A.

[197] Rana N., Suliman S., Al-Sharabi N., Mustafa K. (2022). Extracellular vesicles derived from primed mesenchymal stromal cells loaded on biphasic calcium phosphate biomaterial exhibit enhanced macrophage polarization. Cells.

[198] Song X., Xue Y., Fan S., Hao J., Deng R. (2022). Lipopolysaccharide-activated macrophages regulate the osteogenic differentiation of bone marrow mesenchymal stem cells through exosomes. PeerJ.

[199] Li S.-R., Li D.-W., Man Q.-W. (2024). Proteomic profile of tissue-derived extracellular vesicles from benign odontogenic lesions. J. Stomatol. Oral Maxillofac. Surg..

[200] Du Y., Tang H., Gu X., Shi Y., Gong P., Yao Y. (2021). Radiation can regulate the expression of miRNAs associated with osteogenesis and oxidation in exosomes from peripheral blood plasma. Oxidative Med. Cell. Longev..

[201] Lee A., Choi J., Shi S., He P., Zhang Q., Le A. (2023). DPSC-derived extracellular vesicles promote rat jawbone regeneration. J. Dent. Res..

[202] Liu A., Jin S., Fu C., Cui S., Zhang T., Zhu L., Wang Y., Shen S.G., Jiang N., Liu Y. (2020). Macrophage-derived small extracellular vesicles promote biomimetic mineralized collagen-mediated endogenous bone regeneration. Int. J. Oral Sci..

[203] Ming L., Qu Y., Wang Z., Dong L., Li Y., Liu F., Wang Q., Zhang D., Li Z., Zhou Z. (2024). Small extracellular vesicles laden oxygen-releasing thermosensitive hydrogel for enhanced antibacterial therapy against anaerobe-induced periodontitis alveolar bone defect. ACS Biomater. Sci. Eng..

[204] Shi Y., Zhang R., Da N., Wang Y., Yang J., Li B., He X. (2023). Aspirin loaded extracellular vesicles inhibit inflammation of macrophages via switching metabolic phenotype in periodontitis. Biochem. Biophys. Res. Commun..

[205] Yu W., Li S., Guan X., Zhang N., Xie X., Zhang K., Bai Y. (2022). Higher yield and enhanced therapeutic effects of exosomes derived from MSCs in hydrogel-assisted 3D culture system for bone regeneration. Biomater. Adv..

[206] Zhang B., Pei Z., He W., Feng W., Hao T., Sun M., Yang X., Wang X., Kong X., Chang J. (2024). 3D-printed porous zinc scaffold combined with bioactive serum exosomes promotes bone defect repair in rabbit radius. Aging.

[207] Zhou H., Qi Y.-X., Zhu C.-H., Li A., Pei D.-D. (2023). Mesenchymal stem cell-derived extracellular vesicles for treatment of bone loss within periodontitis in pre-clinical animal models: A meta-analysis. BMC Oral Health.

[208] Wiest E.F., Zubair A.C. (2025). Generation of Current Good Manufacturing Practices-Grade Mesenchymal Stromal Cell-Derived Extracellular Vesicles Using Automated Bioreactors. Biology.

[209] Huang J., Chen H., Li N., Liu P., Yang J., Zhao Y. (2025). Emerging technologies towards extracellular vesicles large-scale production. Bioact. Mater..

[210] Liu X.-C., Tian J.-W., Xu J.-Y., Chen L.-G., Ye Z.-W., Huang J., Wu L.-Z., Zhang Z.-L., Yu Z.-L., Chen G. (2025). Extracellular vesicle manufacture via FACTORY: Fully automated collection technology and optimum machinery for clinical translational applications. Trends Biotechnol..

[211] Singh M., Tiwari P.K., Kashyap V., Kumar S. (2025). Proteomics of Extracellular Vesicles: Recent Updates, Challenges and Limitations. Proteomes.

[212] Silva T.F., Hutchins E., Zhao W., Ciani Y., Kim M., Ko E., Mariscal J., Qiu Z., Bedier F., Kittel A. (2025). Extracellular vesicle heterogeneity through the lens of multiomics. Cell Rep. Med..

[213] Shaba E., Vantaggiato L., Governini L., Haxhiu A., Sebastiani G., Fignani D., Grieco G.E., Bergantini L., Bini L., Landi C. (2022). Multi-omics integrative approach of extracellular vesicles: A future challenging milestone. Proteomes.

[214] Wang T., Ng C.Y., Ng B.Z.J., Toh W.S., Hui J.H.P. (2025). Multi-Omics Analysis of Small Extracellular Vesicles in Osteoarthritis: Bridging the Gap between Molecular Insights and Clinical Applications. Burn. Trauma.

[215] Gómez-de-Mariscal E., Maška M., Kotrbová A., Pospíchalová V., Matula P., Munoz-Barrutia A. (2019). Deep-learning-based segmentation of small extracellular vesicles in transmission electron microscopy images. Sci. Rep..

[216] Uthamacumaran A., Abdouh M., Sengupta K., Gao Z.-h., Forte S., Tsering T., Burnier J.V., Arena G. (2023). Machine intelligence-driven classification of cancer patients-derived extracellular vesicles using fluorescence correlation spectroscopy: Results from a pilot study. Neural Comput. Appl..

[217] del Real Mata C., Jeanne O., Jalali M., Lu Y., Mahshid S. (2023). Nanostructured-Based Optical Readouts Interfaced with Machine Learning for Identification of Extracellular Vesicles. Adv. Healthc. Mater..

